# Identifying and predicting fast versus slow Parkinson’s disease motor progressors using clinical and digital data

**DOI:** 10.1136/bmjno-2026-001740

**Published:** 2026-07-01

**Authors:** Timothee Aubourg, Katarina M Gunter, Christine Lo, Jessica Welch, Karolien Groenewald, Johannes C Klein, Jamil Razzaque, Ludo Van Hillegondsberg, Pietro Luca Ratti, Adriana Nastasa, Grace Auld, Rachel Mccomish, Alexa King, Kashfia Chowdhury, Nirosen Vijiaratnam, Christine Girges, Abigail Patrick, Jemma Inches, Camille B Carroll, Thomas Foltynie, Siddharth Arora, Michele T. Hu

**Affiliations:** 1 Nuffield Department of Clinical Neurosciences, University of Oxford, Oxford, UK; 2 Oxford Parkinson’s Disease Centre, Oxford, UK; 3 Department of Clinical Neurology, Sheffield Teaching Hospitals NHS Foundation Trust, Sheffield, UK; 4 Comprehensive Clinical Trials Unit, University College London, London, UK; 5 Department of Clinical and Movement Neurosciences, University College London Queen Square Institute of Neurology, London, UK; 6 University Hospitals Plymouth, Plymouth, UK; 7 Translational and Clinical Research Institute, Newcastle University, Newcastle upon Tyne, UK; 8 Said Business School, University of Oxford, Oxford, UK; 9 Somerville College, University of Oxford, Oxford, UK

**Keywords:** MOVEMENT DISORDERS, PARKINSON'S DISEASE, CLINICAL NEUROLOGY

## Abstract

**Background:**

Digital health technologies offer high-frequency, objective measures for Parkinson’s disease (PD) yet individual stratification is lacking. This study aimed to identify and predict fast versus slow PD motor progressors by integrating longitudinal clinical evaluation with smartphone motor testing.

**Methods:**

In this 96-week subgroup analysis of participants in the Exenatide-PD3 trial, motor progression was measured using Movement Disorder Society-Unified Parkinson’s Disease Rating Scale Part III OFF scores and smartphone testing (baseline, weeks 24, 48, 72, 96). Fast versus slow progressors were identified using data-driven subtyping; linear mixed-effects models assessed longitudinal OFF scores. Baseline clinical and digital features (in-clinic and at-home) were used to predict 96-week motor progression. Model performance was quantified by the area under the receiver operating curve (AUC).

**Results:**

Data-driven clustering identified 26.5% (26/98) as fast progressors, with higher baseline OFF scores (+18.16, 95% CI 13.70 to 22.62; adjusted p<0.001) and greater 96-week progression (+8.17, 95% CI 2.75 to 13.60; adjusted p=0.019), undetected by prespecified approaches. Baseline prediction models combining smartphone features, alone or with clinical scores, consistently outperformed the clinical model alone (best AUC=0.78 vs 0.53). Translational value of smartphone assessments was strengthened by high user acceptability (>96% support future in-clinic use) and predictive value in a deployment scenario (AUC 0.80, 95% CI 0.79 to 0.82).

**Conclusion:**

Data-driven clustering identified one in four early-to-mid stage PD patients as fast motor progressors. Integrating smartphone-derived features with clinical scores improved baseline motor prediction. High user acceptability supports this approach for individual-level PD stratification, addressing heterogeneity in motor progression that may obscure treatment trial effects.

**WHAT IS ALREADY KNOWN ON THIS TOPIC**

- Traditional prognostic modelling in Parkinson’s disease relies on clinical rating scales, often failing to capture patient heterogeneity at baseline and progression, leading to false positive or negative treatment effects. While digital tools provide high-frequency data, a practical framework for integrating digital biomarkers with standard clinical assessments for patient-level stratification is lacking.

**WHAT THIS STUDY ADDS**

- We demonstrate that a data-driven clustering approach on longitudinal motor scores identified a substantial subgroup of fast motor progressors (26.5%), invisible to conventional clinical categorisation. Combining smartphone-derived digital features with baseline clinical scores substantially improved 96-week motor progression prediction, outperforming the clinical model alone. In-clinic and at-home digital measures both showed predictive value.

**HOW THIS STUDY MIGHT AFFECT RESEARCH, PRACTICE OR POLICY**

- This study provides a scalable, well-tolerated, digital-clinical phenotyping framework to deliver baseline, patient-level stratification in clinical trials. Implementing this approach will (a) reduce neuroprotective trial numbers needed to treat and related costs, (b) increase understanding of placebo and nocebo effects and (c) support early, personalised medicine deliveries across routine Parkinson’s clinical practice.

## Introduction

 Parkinson’s disease (PD) is the second most common neurodegenerative disorder^1^ and one of the fastest-growing neurological conditions globally.^2^ Motor progression heterogeneity is a major challenge for clinical trials using motor endpoints in PD, increasing the sample sizes and costs needed to detect treatment effects.

Clinical factors, including motor phenotype, age at onset, sex and disease duration have been associated with differential progression patterns and may provide valuable prognostic insights to clinicians.^3^ Although baseline motor severity is a probable predictor of progression rate, the overall prognostic value of many clinical features remains uncertain.^4^

Improved methods and tools for the identification and prediction of PD motor progression are needed. Broadly accepted clinical assessments, such as the Movement Disorder Society-Unified Parkinson’s Disease Rating Scale Part III (MDS-UPDRS-3), provide standardised assessments of motor symptoms.^5^ While informative, their sporadic application offers only limited temporal resolution, is subject to inter-rater variability and may not reflect daily life performance.^1 6 7^ High-frequency digital biomarkers from smartphones and wearables offer ecologically valid, objective motor measures that can mitigate such limitations.^8^,^1010^ Prior studies have demonstrated that digital measures can detect motor abnormalities and correlate with clinical ratings of disease severity in both in-clinic and at-home settings.^12^,^1418^

However, to our knowledge, no study integrates digital biomarkers with clinical features to identify and predict motor progression phenotypes in PD within a single framework. This gap remains a significant barrier to the development of individualised prognostic models and the broader implementation of precision medicine in PD care.

The Exenatide-PD3 trial,^19^ a phase III, multicentre, double-blind, parallel-group, randomised, placebo-controlled study, provides a unique opportunity to investigate integrative, data-driven approaches to monitoring PD progression. In this exploratory analysis, we evaluated 98 participants over a 96-week period, combining standard in-clinic MDS-UPDRS-3 assessments with smartphone-derived motor metrics collected during both clinic visits and at-home testing. Our aim was twofold: (a) to identify faster versus slower motor progressors using prespecified clinical variables (eg, age, sex, motor subtype) alongside data-driven subgrouping and (b) to assess whether baseline clinical and digital biomarkers could improve the prediction of long-term motor trajectories beyond traditional clinical measures alone.

To this end, we developed a data-driven framework following five sequential objectives:

1. Identify subgroups of faster versus slower motor progressors, potentially exhibiting differential responses to exenatide versus placebo.
2. Evaluate whether baseline clinical and smartphone-derived features could predict motor trajectories over 96 weeks based on the subgroups identified in (1).
3. Evaluate whether longitudinal clinical and smartphone-derived features could predict Exenatide-PD3 treatment allocation (placebo vs exenatide).
4. Evaluate the digital smartphone app user experience in a real-life phase III clinical trial.
5. Explore practical real-world deployment of the smartphone app, using a hybrid digital protocol simulation.

### Hypothesis

We hypothesised that integrating smartphone-based motor assessments with conventional clinical evaluations in a unified, data-driven framework would improve the characterisation and early prediction of progression phenotypes.

## Materials and methods

### Study design, setting and participants

This research was a 96-week subgroup analysis of 103 out of 194 participants enrolled in the Exenatide Phase III trial (Exenatide-PD3), a multicentre, double-blind, randomised, placebo-controlled study. The digital substudy recruited participants from three UK trial sites: University College London Hospitals NHS Trust, Oxford University Hospitals NHS Foundation Trust and University Hospitals Plymouth NHS Trust.

**Figure 1:**
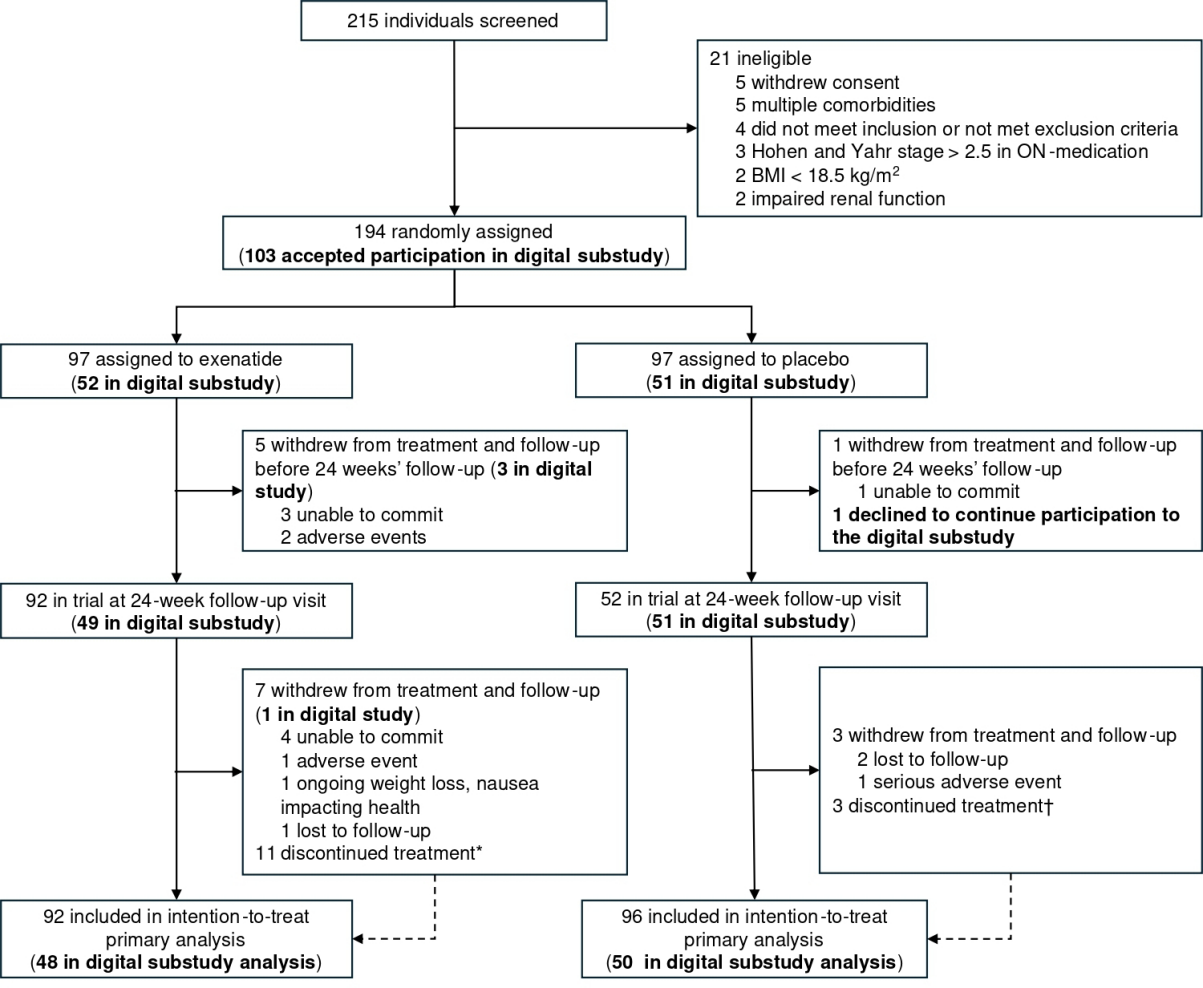
Parent trial and digital substudy selection profile. *Reasons for treatment discontinuation were adverse events (n=8), weight loss and worsening of Parkinson’s disease (n=1), potential product complaint, unsatisfactory response and unwilling to continue (n=1) and increased anxiety regarding comorbid health issues (n=1). †Reasons for treatment discontinuation were adverse events (n=1) and serious adverse events (n=2). For the digital substudy (participants shown in bold), among 103/194 who accepted participation, there were four withdrawals from the exenatide group due to loss to follow-up in the Exenatide-PD3 trial, and one withdrawal from the placebo group due to declining to continue participation in the digital substudy. BMI, body mass index.

The trial design has been fully detailed in *The Lancet*^19^ (online supplemental table 1), with the resulting study profile detailed in figure 1. Briefly, the inclusion criteria required a PD diagnosis, age 25–80 years, Hoehn and Yahr stage ≤2.5 (ON state) and stable dopaminergic treatment for ≥4 weeks. Exclusion criteria included suspicion of atypical Parkinsonism, significant cognitive impairment (Montreal Cognitive Assessment score <21) or severe depression (Patient Health Questionnaire-9 ≥16).

### Clinical assessments

The primary outcome for progression was a change in the MDS-UPDRS-3 OFF score. Motor progression was measured at five visits: baseline, weeks 24, 48, 72 and 96. Clinical assessments were conducted in both OFF (≥8 hours since last short-acting PD medication and ≥36 hours since last long-acting PD medication) and ON states (online supplemental figure 1).

### Smartphone-based assessments

**Figure 2:**
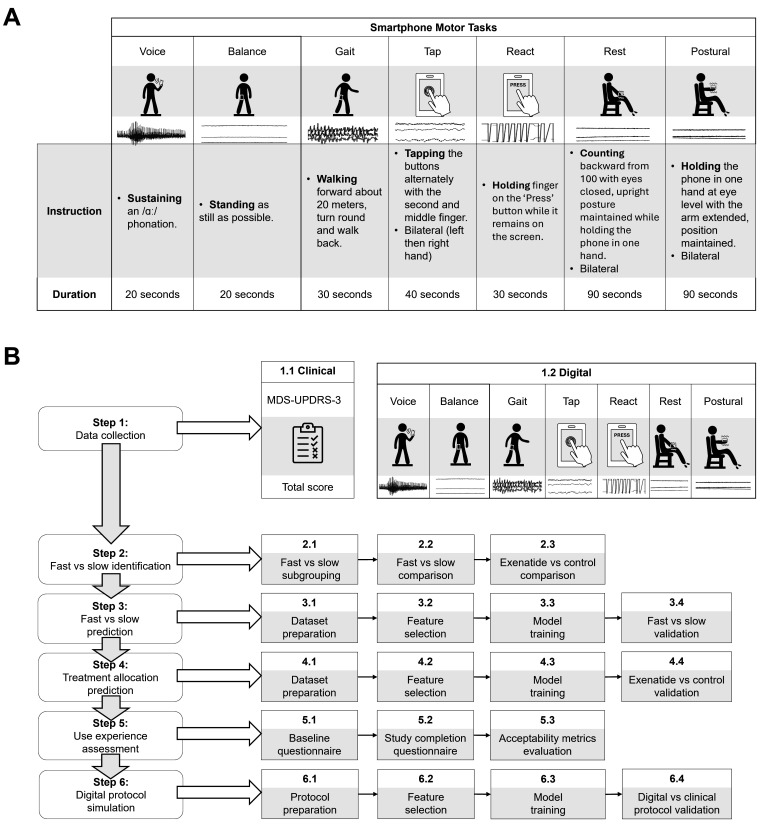
(A) Smartphone motor tasks and participant instructions. (B) Overview of the data analysis workflow. The six-step strategy for evaluating clinical and digital data is illustrated. *Step 1*: collection of clinical and smartphone-based digital records. Reaction time is performed with the dominant hand, and rest tremor, postural tremor and finger tapping tasks are performed with both hands to assess for asymmetry. *Step 2*: classification of participants as fast or slow motor progressors and comparison of Exenatide versus placebo subgroups. *Step 3*: prediction of motor progression at baseline using clinical and/or smartphone-derived features. *Step 4*: prediction of treatment allocation based on smartphone data. *Step 5*: assessment of participant smartphone user experience. *Step 6*: simulation of the digital protocol to evaluate motor progression prediction at baseline under digital versus clinical scenarios. MDS-UPDRS-3, Movement Disorder Society-Unified Parkinson’s Disease Rating Scale Part III.

The smartphone protocol^8^ consisted of a 7-minute battery of seven tasks: voice, balance, gait, finger tapping, reaction time, rest tremor and postural tremor (see figure 2). Tasks captured kinematic and audio data via smartphone sensors via tri-axial accelerometer, gyroscope, with data encrypted and transmitted via Wi-Fi. In-clinic assessments were clinician-supervised. Participants also performed at-home assessments three times per day for 1 week following each 6-month clinic visit using loaned smartphones, yielding a maximum of 105 assessments per participant.

### Statistical analysis

#### Identification of faster versus slower motor progressors

Participants were classified as *faster* or *slower* progressors using two methods:

1. Prespecified subgrouping, based on threshold classification over seven prespecified clinical variables (age, sex, disease duration, motor phenotypes I and II, Hoehn and Yahr stage), following the approach developed by Pagano *et al*^20^ (online supplemental table 2).
2. Data-driven subgrouping, based on longitudinal MDS-UPDRS-3 OFF clustering using k-means on pairwise distances calculated with dynamic time warping (DTW), where DTW is a method for comparing time series that may evolve at different speeds or with misalignments in time, by flexibly *warping* the time axis to align similar patterns (online supplemental table 2). As part of a supplementary post-hoc analysis, we compared four different clustering analysis approaches to the above DTW binary classification of fast versus slow motor progressors (see online supplemental material, post-hoc analysis, for details).

Linear mixed-effects models (LMMs) for repeated measures assessed changes in MDS-UPDRS-3 OFF scores from baseline to week 96 between faster and slower groups across each subgrouping scenario. P values were adjusted for multiple comparisons using the Benjamini-Hochberg false discovery rate method. Only significant subgrouping scenarios were retained for the next part of the analysis on motor trajectory prediction. LMMs were also employed to compare exenatide versus placebo effects within each subgroup. Treatment effects were reported as both absolute differences in mean change and relative difference (RD) ratios, calculated as the mean change difference over the placebo group’s mean change.

#### Prediction of *faster* versus *slower* motor progressors at baseline

Baseline predictions of faster versus slower motor progressors, as determined in the previous subgrouping analyses (objective 1), were performed using five classifier models to capture different assessment modalities and real-world clinical scenarios: (a) clinical benchmark (MDS-UPDRS-3 OFF), (b) smartphone-only (in-clinic data), (c) smartphone-only (in-clinic with at-home data), (d) multimodal (MDS-UPDRS-3 with in-clinic smartphone data), (e) multimodal (MDS-UPDRS-3 with in-clinic and at-home smartphone data).

#### Prediction of treatment allocation

To assess the consistency of the digital data with the Exenatide-PD3 trial’s negative primary outcome,^19^ we evaluated whether clinical and smartphone-derived features could predict treatment assignment (exenatide vs placebo). Classifiers were applied using three models: (a) clinical benchmark (MDS-UPDRS-3 OFF), (b) smartphone (in-clinic), (c) smartphone (in-clinic and at-home data). Models were expected to perform near random (area under the receiver operating curve (AUC) ≈0.5), serving as a sanity check to confirm the lack of treatment effect previously reported by the trial.

#### Evaluation of smartphone user experience

Furthermore, we quantified the feasibility and reliability of test completion using two metrics: compliance (≥7 tests/week) and adherence (very high: ≥3 tests/day; high: 2–3/day; moderate: 1–2/day; low: ≤1/day), calculated each week following each clinic visit (online supplemental table 4 and figure 2).

#### Digital protocol simulation

To simulate real-world trial deployment with heterogeneous data availability, we compared two protocol strategies for classifying the entire trial population (n=98, online supplemental figure 3):

1. Clinical protocol (single-pathway): all participants were classified using the MDS-UPDRS-3 alone model.
2. Digital protocol (dual-pathway): participants with available baseline smartphone data were classified using the multimodal model (clinical+digital features); those without were classified using the MDS-UPDRS-3 alone model. Predictions from both pathways were aggregated for a single protocol-level outcome.

This simulation allowed us to test whether integrating smartphone data (digital protocol) improves predictive performance at the population level compared with a clinical-only approach (clinical protocol).

### Machine learning procedure

Predictive modelling for objectives 2, 3 and 5 was implemented using a standardised supervised machine learning workflow in Python (V.3.10.0), ensuring unbiased evaluation through leave-one-out cross-validation (LOO-CV). All preprocessing, feature selection, and model training steps were strictly nested within the LOO-CV loop to prevent data leakage and provide a conservative assessment of generalisability.

#### Feature engineering, selection and missing data

Smartphone feature vectors were extracted following established methodologies for PD digital biomarkers.^13 14^ Regarding missing data, strict inclusion criteria were applied at the data-point level, requiring complete MDS-UPDRS-3 OFF/ON scores, a full set of smartphone features and temporal alignment (at-home data within 1 week of the clinic visit). To minimise overfitting, a nested feature selection procedure was performed independently within each LOO-CV training fold. This involved:

1. Generating candidate feature subset grid (10–300 features).
2. Selecting the optimal subset size based on the maximal Youden’s J statistic on the training set.
3. Training the classifier using the features from the top-ranked selected subset.

#### Model training and evaluation

Tree-based algorithms were used in the LOO-CV:

1. Clinical benchmark model: a decision tree classifier was used, as it included only a single feature (MDS-UPDRS-3 OFF score).
2. Smartphone-based and multimodal models: random forest classifiers were employed for multidimensional data. For models using multiple at-home recordings, an ensemble prediction strategy was implemented, averaging individual prediction probabilities to achieve the final participant-level outcome. To prevent data leakage, the optimal probability threshold for binary classification was determined independently within the training fold using Youden’s J statistic, then applied to the test fold.

Model performance was quantified using the Area Under the Receiver Operating Characteristic Curve (AUC). To assist clinical application, additional metrics, including sensitivity, specificity, positive predictive value (PPV), and negative predictive value (NPV) and Brier score were reported in online supplemental file.

Data analyses were primarily focused on MDS-UPDRS-3 OFF scores and were repeated for ON state (online supplemental tables 5–14, figures 4–6). For analyses involving smartphone features, only participants who completed smartphone testing in both ON and OFF states at baseline were included (n=47) to ensure consistency across the analyses.

## Results

### Baseline characteristics

Of the 103 participants enrolled in the Exenatide-PD3 digital substudy, five withdrew (four lost to follow-up within the main trial; one declined continued digital participation), leaving 98 for analysis (figure 1).

The cohort reflected early- to mid-stage PD: 88.8% had Hoehn and Yahr stage ≤2, mean (SD) age was 61.5 (8.7) years, 77.6% were male and 94.9% self-identified as white, with baseline characteristics balanced between treatment groups (table 1).

**Table 1:** Baseline demographic and clinical characteristics of the study population

Characteristic	Population	Exenatide	Placebo
Participants, n (%)	98 (100)	48 (49)	50 (51)
Age at baseline, mean (SD), years	61.5 (8.7)	62.0 (8.5)	61.0 (9.0)
(95% CI)	(60.9 to 62.0)	(59.6 to 64.5)	(58.5 to 63.6)
Age at diagnosis, mean (SD), years	57.0 (9.3)	57.2 (9.3)	56.9 (9.4)
(95% CI)	(55.2 to 58.9)	(54.5 to 59.9)	(54.2 to 59.5)
Disease duration at baseline, mean (SD), months	54.1 (33.4)	58.5 (37.5)	49.9 (28.6)
(95% CI)	(47.4 to 60.8)	(47.6 to 69.4)	(41.8 to 58.1)
BMI, mean (SD)	25.9 (4.2)	26.7 (4.5)	25.2 (3.8)
(95% CI)	(25.1 to 26.8)	(25.4 to 28.0)	(24.2 to 26.3)
Height, mean (SD), cm	175.8 (9.0)	175.9 (8.7)	175.7 (9.3)
(95% CI)	(174.0 to 177.6)	(173.3 to 178.4)	(173.0 to 178.3)
Weight, mean (SD), kg	80.2 (14.7)	82.6 (15.5)	77.9 (13.5)
(95% CI)	(77.2 to 83.1)	(78.1 to 87.1)	(74.0 to 81.7)
Sex, n (%)			
Female	22 (22.4)	9 (18.8)	13 (26.0)
Male	76 (77.6)	39 (81.2)	37 (74.0)
Ethnicity, n (%)			
White	93 (94.9)	47 (97.9)	46 (92.0)
Asian and other	5 (5.1)	1 (2.1)	4 (8.0)
Hoehn and Yahr stage, n (%)			
≤2	87 (88.8)	44 (91.7)	43 (86.0)
>2	11 (11.2)	4 (8.3)	7 (14.0)

BMI, body mass index.

At-home smartphone assessments were completed by 78 participants (80%) in the first week, and 74 (76%) met the compliance threshold (≥7 test/weeks) (online supplemental figure 2 and table 4). In-clinic smartphone testing in both OFF and ON states was completed by 47 participants (48%) at baseline.

### Identification of *fast* versus *slow* motor progressors

#### Data-driven trajectories separate progressor groups most clearly

**Figure 3:**
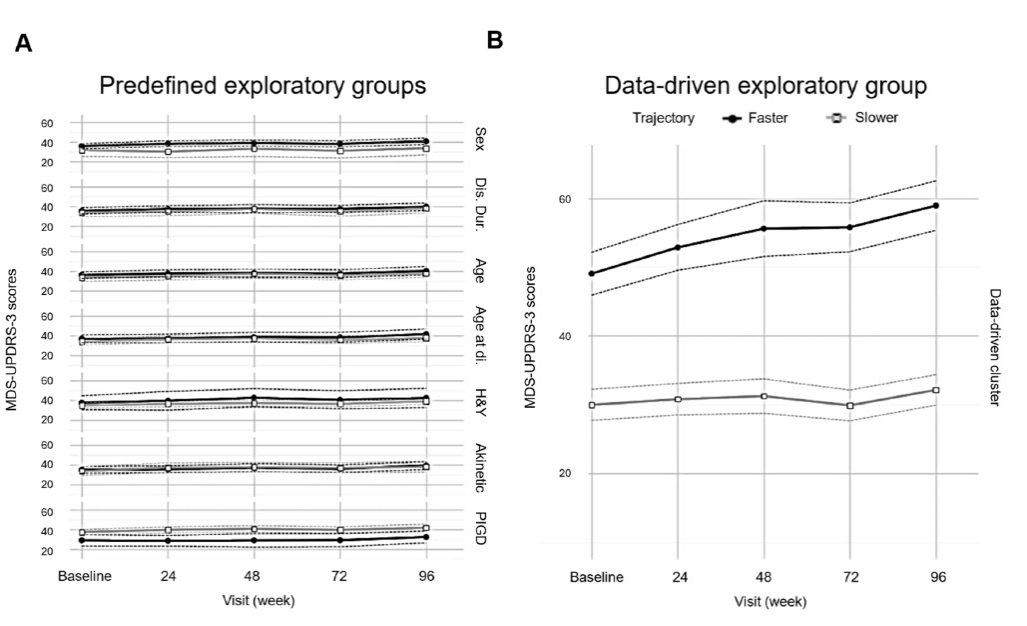
MDS-UPDRS-3 scores mean and 95% CI across clinical visits for slower and faster subgroups for (A) predefined exploratory subpopulations: sex, disease duration, age at baseline, age at diagnosis, Hoehn and Yahr stage, akinetic-rigid and PIGD motor phenotypes and (B) data-driven exploratory subpopulations. MDS-UPDRS-3, Movement Disorder Society-Unified Parkinson’s Disease Rating Scale Part III; PIGD, postural instability and gait difficulty.

LMMs showed that the data-driven clustering identified 26.5% (26/98) as fast progressors, with higher baseline OFF scores (+18.16, 95% CI 13.70 to 22.62; adjusted p<0.001) and greater 96 week progression (+8.17, 95% CI 2.75 to 13.60; adjusted p=0.019) (figure 3, table 2).

**Table 2:** Movement Disorder Society-Unified Parkinson’s Disease Rating Scale Part III OFF scores across data-driven subpopulations

Parameter	Coefficient	95% CI	Z	P value	Adjusted p value
Intercept	27.65	21.56 to 33.74	8.894	<0.001	<0.001
Change, baseline to week 96 (set to slower groups)	2.78	−4.63 to 10.19	0.735	0.463	0.891
Data-driven cluster *fast* versus *slow*	18.16	13.70 to 22.62	7.979	<0.001	<0.001
Additional change from baseline to week 96, data-driven cluster *fast* versus *slow*	8.17	2.75 to 13.60	2.953	0.003	0.019

Prespecified clinical subgroups showed only small, non-significant differences in progression (eg, +1.59 points for older age at diagnosis; +2.34 for males; online supplemental table 5).

##### Greater deterioration in exenatide data-driven fast progressors

Among data-driven fast progressors, those receiving exenatide showed greater worsening than placebo (table 3, online supplemental table 8). Mean OFF scores increased from 51.54 to 57.31 in the placebo group and from 46.54 to 60.54 in the exenatide group, yielding an adjusted mean change of 5.77 versus 14.00 points (95% CI, 5.91 to 10.55; RD, 1.43). The exploratory framing of this analysis precludes a causal interpretation at this stage.

**Table 3:** Exenatide effects on motor progression as measured by the Movement Disorder Society-Unified Parkinson’s Disease Rating Scale Part III OFF score across the predefined and data-driven exploratory subpopulations

Category	Subgroup	Trajectory	Total n	Adjusted-mean ($\Delta {\;}_{baseline\to Week96}$)			95% CI	Relative difference (RD) ratio
				Placebo	Exenatide	Difference		
Sex	Female	Slower	22	0.77	3.78	3.01	2.02 to 4.00	3.91
	Male	Faster	76	2.54	7.05	4.51	3.68 to 5.34	1.78
Disease duration	≤48 months	Slower	39	2.33	6.67	4.33	0.44 to 8.23	1.86
	>48 months	Faster	59	1.90	6.30	4.40	4.09 to 4.72	2.32
Age	≤60 years	Slower	39	1.38	7.44	6.06	4.03 to 8.10	4.39
	>60 years	Faster	59	2.59	5.83	3.25	1.84 to 4.65	1.26
Age at diagnosis	≤60 years	Slower	60	2.23	5.69	3.46	2.26 to 4.66	1.56
	>60 years	Faster	38	1.84	7.58	5.74	4.59 to 6.88	3.11
Hoehn and Yahr stage	≤2	Slower	87	1.81	6.43	4.62	3.49 to 5.75	2.55
	>2	Faster	11	3.71	6.50	2.79	−3.95 to 9.52	0.75
Motor phenotypes I	Tremor-dominant	Slower	23	2.80	6.88	4.08	3.30 to 4.85	1.46
	Akinetic-rigid	Faster	64	2.29	4.00	1.71	−0.92 to 4.34	0.75
Motor phenotypes II	Tremor-dominant	Slower	72	2.00	7.09	5.09	0.48 to 9.70	2.55
	PIGD	Faster	20	3.00	5.81	2.81	1.97 to 3.66	0.94
Data-driven motor trajectory	Slow	Slower	72	0.78	3.63	2.84	1.51 to 4.18	3.63
	Fast	Faster	26	5.77	14.00	8.23	5.91 to 10.55	1.43

PIGD, postural instability and gait difficulty.

Among data-driven slow progressors, changes were smaller (placebo: +0.78; exenatide: +3.63; difference 2.84; 95% CI 1.51 to 4.18). Across prespecified clinical subgroups, exenatide–placebo differences were smaller and inconsistent, and no clear pattern of treatment benefit emerged (table 3, online supplemental table 8).

### Smartphone-based assessments improve baseline prediction of motor progression

Smartphone-only models outperformed the MDS-UPDRS-3 benchmark (table 4). The conventional clinical model showed discrimination akin to chance level (AUC 0.53; 95% CI 0.47 to 0.59). In-clinic smartphone-derived features markedly improved classification (AUC 0.78; 95% CI 0.72 to 0.82). Adding MDS-UPDRS-3 scores provided similar accuracy (AUC 0.76; 95% CI 0.71 to 0.81). A multimodal model combining in-clinic and at-home data yielded comparable results (AUC 0.76; 95% CI 0.72 to 0.82).

**Table 4:** Predictive performance of classifiers for fast versus slow motor progression trajectories at baseline

Classifier configuration	AUC (95% CI)
Benchmark (MDS-UPDRS-3 only)	0.53 (0.47 to 0.59)
Smartphone-only (in-clinic data)	0.78 (0.72 to 0.82)
Multimodal-model (MDS-UPDRS-3+in-clinic smartphone data)	0.76 (0.71 to 0.81)
Smartphone-only (in-clinic and at-home data)	0.70 (0.67 to 0.72)
Multimodal-model (MDS-UPDRS-3+in-clinic and at-home smartphone data)	0.76 (0.72 to 0.82)

AUC, area under the receiver operating curve; MDS-UPDRS-3, Movement Disorder Society-Unified Parkinson’s Disease Rating Scale Part III.

Feature-selection analysis highlighted rest tremor, gait and postural tremor as the most predictive tasks, with rest tremor features selected most frequently (mean selection frequency 39.7%, SD 5.51%; online supplemental figure 5, tables 9–12).

### Smartphone and clinical features do not predict treatment allocation

Classifiers trained to distinguish exenatide from placebo performed at chance level (AUC≈0.5; online supplemental table 13), consistent with the negative primary outcome of the Exenatide-PD3 trial.^19^

### Adherence and acceptability of smartphone assessments

Adherence to at-home testing varied across 96 weeks (online supplemental table 4, figure 2). Median test counts declined modestly from 18 at baseline to 10.5 at week 96. Compliance (≥7 weekly tests) remained high throughout (79%, 71%, 63% and 58% at weeks 24, 48, 72 and 96). In-clinic assessments increased to 66 participants (67%) by week 72, and only one participant did not complete any smartphone testing.

Questionnaire responses (84/98 at baseline, 77/98 at endpoint) indicated strong acceptability: 92% rated the app easy to use, 89% endorsed home use and 96% supported in-clinic use.

### Digital protocol evidenced predictive value

The digital protocol outperformed the MDS-UPDRS-3 clinical-only approach (online supplemental table 14), achieving a higher predictive accuracy (AUC 0.80, 95% CI 0.79 to 0.82 vs AUC 0.72, 95% CI 0.70 to 0.74).

## Discussion

We integrated clinical assessments with brief smartphone-based motor measures in a data-driven framework to identify and predict motor-progression phenotypes within the Exenatide-PD3 cohort. As expected, classification from prospective serial motor scores outperformed baseline clinical characteristics in separating progression trajectories. A novel finding is that baseline smartphone features showed predictive value for 96-week motor progression beyond the MDS-UPDRS-3 benchmark.

Our data-driven clustering classified approximately one in four participants as fast versus slow progressors (26.5% vs 73.5%), a distinction not captured by prespecified clinical subgroup comparisons (eg, age, sex, motor phenotype). The overall motor progression rate (~1.8% per annum of the 132-point maximum MDS-UPDRS-III) was consistent with the 2.1% reported in the full Exenatide-PD3 cohort.^19^ Notably, placebo-arm progression rates diverged markedly between subgroups—slow progressors at ~0.3% p.a. versus fast progressors at ~2.4% p.a. (table 3)—underscoring the clinical relevance of identifying these trajectories early.

### Data-driven stratification reveals clinically relevant heterogeneity

Conventional baseline characteristics failed to reliably separate progression trajectories (figure 3), reflecting known PD heterogeneity.^3 4 21^ The data-driven trajectories captured a subset with higher baseline MDS-UPDRS-3 OFF scores and markedly steeper 96-week decline, defining a clinically distinct fast-progression subgroup. This subgroup showed the largest divergence between treatment arms—fast progressors on exenatide worsened more than placebo-treated fast progressors—an exploratory finding illustrating how data-driven stratification can reveal heterogeneity that may distort treatment signals in conventional analyses.

These results suggest that conventional baseline clinical stratification alone is insufficient for precision enrichment in neuroprotective trials. Data-driven approaches add discriminative power to identify participants at elevated near-term risk of progression, permitting more efficient designs and smaller samples, critical where annual progression is modest and placebo/nocebo effects mask true benefit.

### Digital measures and clinical translation

A central challenge in PD therapeutics is identifying neuroprotective treatments that slow progression at an individual, personalised level. Exenatide-PD3 rigorously demonstrated that exenatide did not alter the rate of PD progression.^19^ Yet despite major advances in understanding PD pathobiology, no trial to date has demonstrated disease modification, a central obstacle being the substantial heterogeneity in baseline features and progression across motor, non-motor and cognitive domains. We previously demonstrated this in a data-driven subtyping study across the Oxford Discovery and Tracking Parkinson’s cohorts (n=2545 early PD), which identified four clusters with a 3.0-point/year difference in MDS-UPDRS-3 progression between the slowest and fastest progressors, equivalent to a clinically meaningful trial endpoint.^22 23^ Baseline stratification in neuroprotective trials nonetheless remains limited,^24^ underscoring the need for better methods to identify those most likely to benefit and to enable earlier go/no-go decisions.

Smartphone-based digital measures can help address this gap. By capturing bilateral tremor, gait, postural stability and reaction-time performance through a brief, low-burden protocol, they provide objective, granular information about baseline motor physiology. Feature selection indicated rest-tremor and gait features were most predictive of clinically significant progression, consistent with evidence linking axial and gait dysfunction to faster decline.^25^,^27^ The prominence of tremor features, despite tremor not contributing to postural instability and gait difficulty/axial scoring, aligns with Parkinson’s progression markers initiative data on early PD presentation.^28^ Reaction-time features were selected more often in OFF states (online supplemental figure 6), potentially reflecting levodopa-sensitive attentional mechanisms.

Crucially, integrating clinical data with brief smartphone assessments enabled scalable remote stratification beyond conventional subgrouping, with combined clinical–digital models outperforming clinical-only models at baseline, extending our recent finding that single-test smartphone features predicted striatal dopamine status with 85% accuracy alongside MDS-UPDRS-3 scores.^29^

### Limitations and future directions

These findings should be interpreted as exploratory and hypothesis-generating. The modest sample size and reduced diversity (94.9% White; 77.6% male) may limit generalisability. The binary fast-versus-slow classification was retained for consistency with the prespecified subgrouping framework,^20^ with post-hoc validation (online supplemental material) supporting its stability; more granular clustering could be explored in future work, in dialogue with existing PD subgroupings.^22 30^

The cohort was drawn from three UK sites under a standardised protocol, so site-level differences may affect model transportability and external validation across independent centres remains necessary. Smartphone participation also required reliable Wi-Fi, potentially introducing selection bias by excluding those with limited connectivity and more inclusive deployment strategies will be important for future studies. Finally, although user acceptability was high, engagement with repeated remote assessments decayed progressively over the 96 weeks, possibly compounded by COVID-19-era disruptions; future studies should examine the relationship between acceptability and sustained engagement, and the specificity of digital health-technology engagement in both clinical-trial and real-world settings.

### Conclusions

Integrating smartphone assessments with clinical measures identified and predicted distinct motor-progression trajectories in PD: data-driven clustering revealed that 26.5% of participants followed fast-progression trajectories, undetectable by conventional subgrouping. Baseline digital biomarkers with in-clinic measures achieved up to 80% accuracy in predicting 96-week progression. Although patient-level deployment will require validation in larger, more diverse independent cohorts, this approach offers a practical route to personalised baseline stratification and to clinical prognostication from first specialist referral or diagnosis.

## Supplementary material

10.1136/bmjno-2026-001740 online supplemental file 1

## Data Availability

Data are available upon reasonable request.
