# Supplementary material for: Identifying and predicting fast versus slow Parkinson’s disease motor progressors using clinical and digital data
Source: BMJ Neurol Open. 2026 Jul 1;8(2):e001740. doi: 10.1136/bmjno-2026-001740 (PMC13331057; doi:10.1136/bmjno-2026-001740)
Supplement: online supplemental file 1 [file bmjno-8-2-s001.docx]

# **Supplementary material**

**Number of Figures**:7, **Number of Tables**: 22, **Number of Appendix subsections**: 1 (Results).


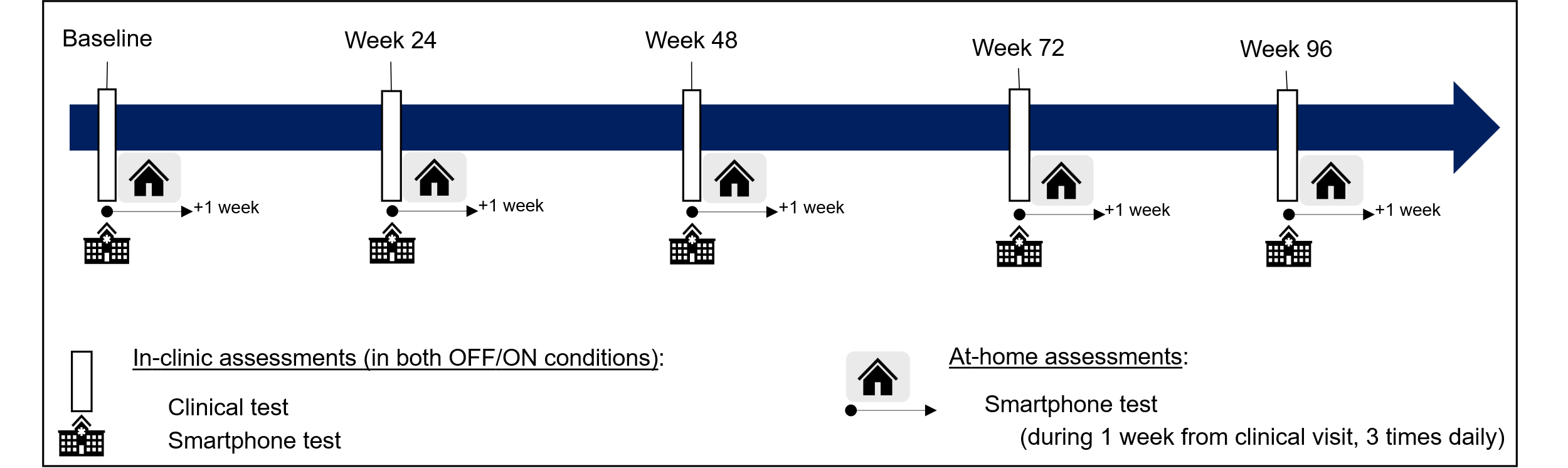
Figure 1. Overview of the data acquisition protocol.


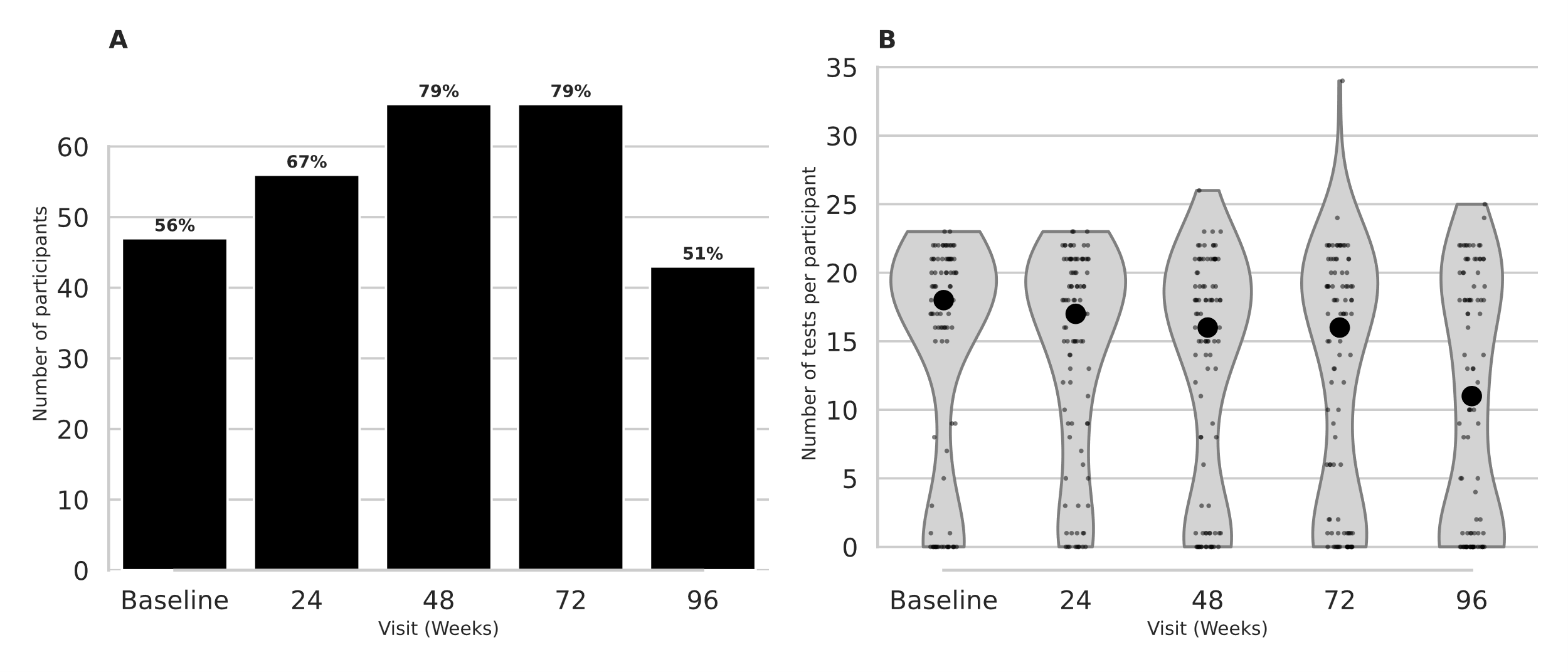


Figure 2. Participant smartphone test completion across visits: in-clinic and at-home assessments. **Panel A** shows the number of participants who completed the smartphone test in clinic. **Panel B** shows the distribution of the number of at-home assessments in the week following the clinical visit. These metrics were summarized using number and proportion of participants meeting the compliance threshold at each visit, to inform feasibility and reliability of smartphone-based assessments in the clinical trial setting.


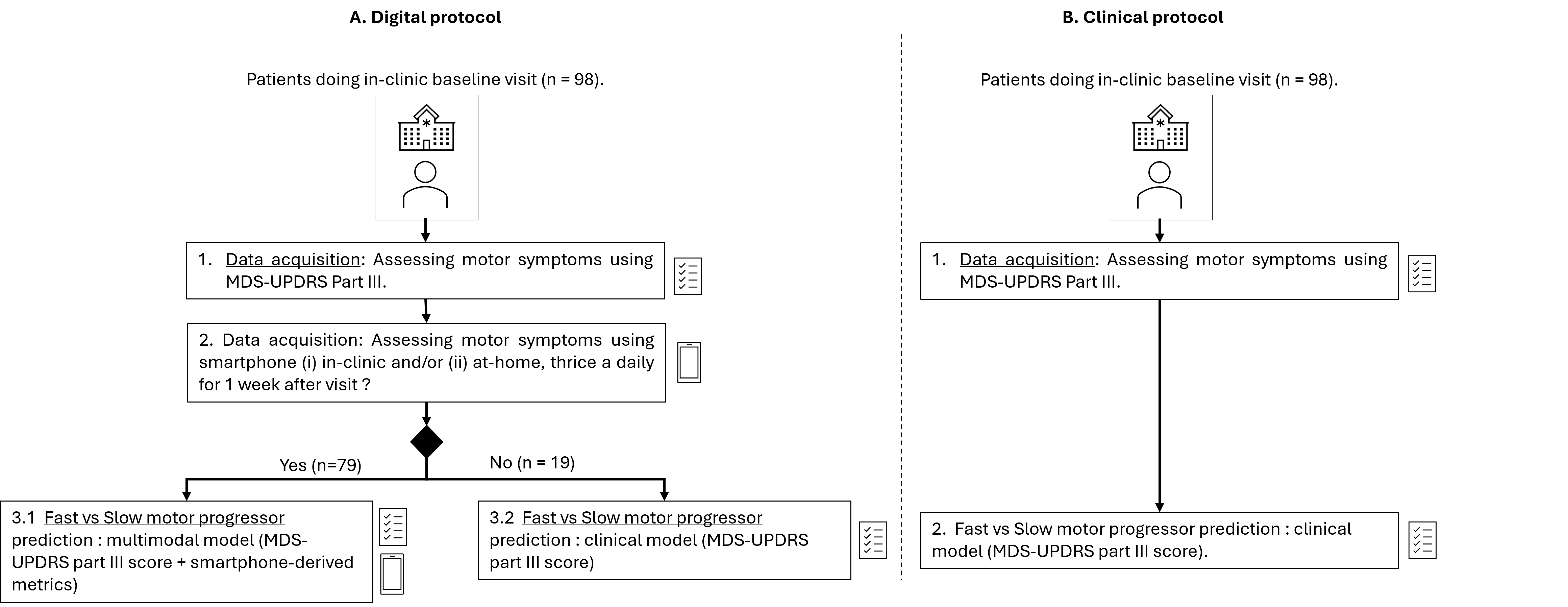


Figure 3. Comparison of digital and conventional protocols for baseline prediction of Parkinson’s disease motor trajectory. **Panel A** depicts a digital protocol, where participants complete smartphone-based assessments alongside the MDS-UPDRS-3 evaluation. Predictions are generated using either (i) a multimodal model integrating clinical scores and smartphone data or (ii) a clinical model relying solely on clinical scores, based on data availability. **Panel B** illustrates a clinical conventional protocol, where only MDS-UPDRS-3 scores are used for prediction.

**
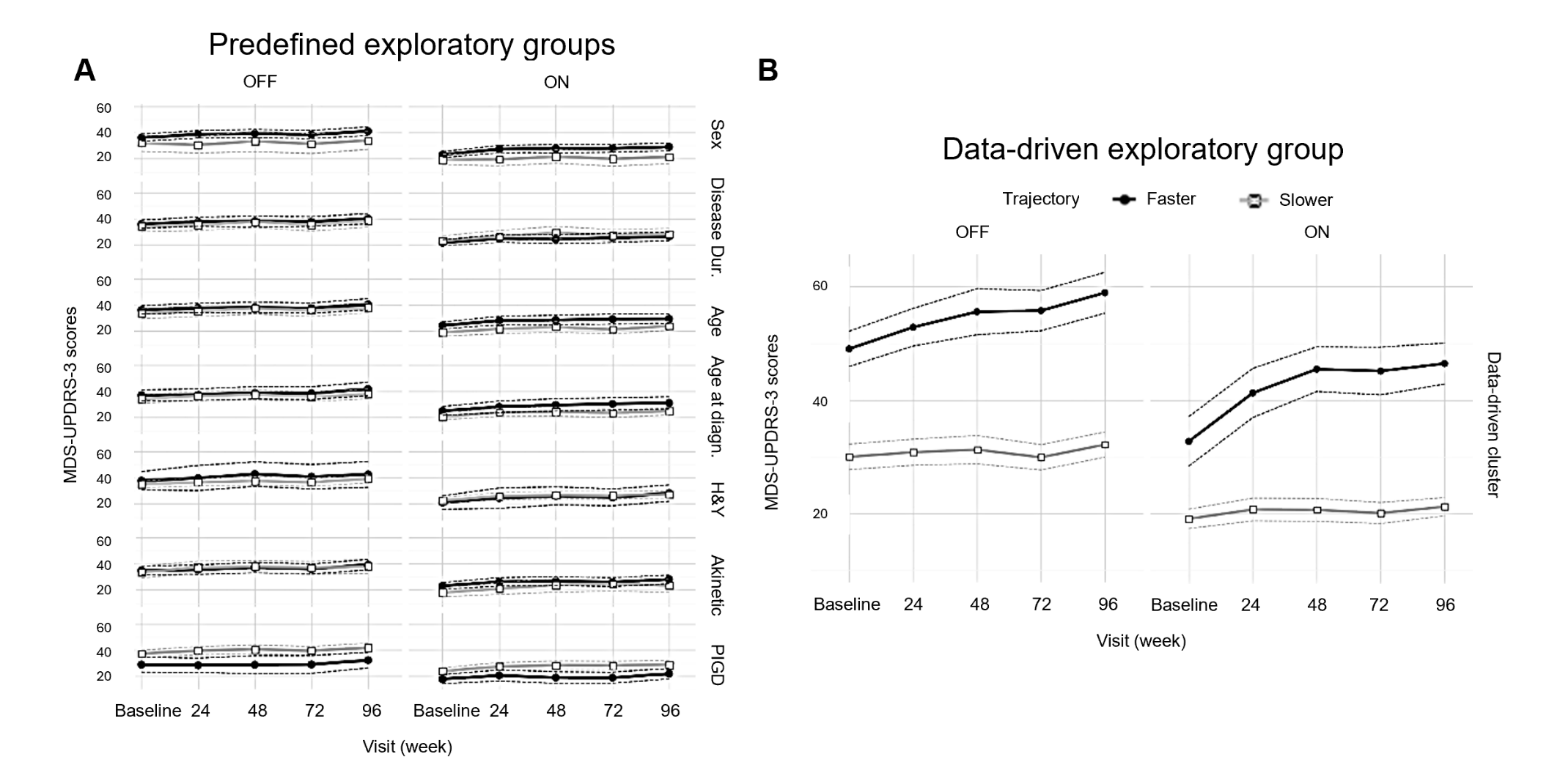
**

Figure 4. MDS-UPDRS-3 scores mean and 95%CI across clinical visits for slower and faster subgroups in both OFF and ON conditions **for: (Panel A)** predefined exploratory subpopulations: sex, disease duration, age at baseline, age at diagnosis, Hoehn and Yahr stage, Akinetic-rigid and PIGD motor phenotypes, and **(Panel B)** data-driven exploratory subpopulations.


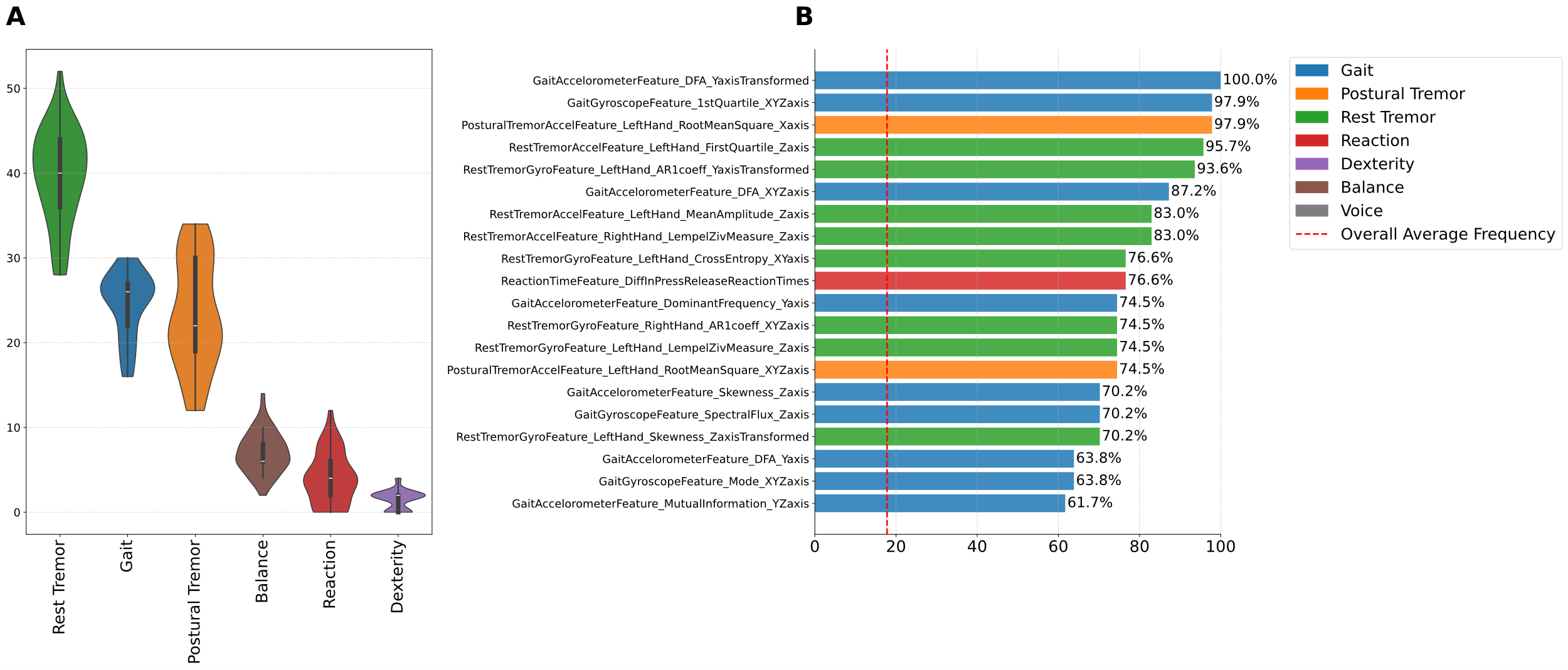


Figure 5. Feature selection summary across smartphone assessments in OFF-medication state. **Panel A** reports violin plot of the proportion of features selected per motor task across leave-one-out cross-validation folds. **Panel B** reports horizontal bar plot of the selection frequency (%) of the top 20 individual features; bars are colored by motor task, and the red dashed line indicates the overall average feature selection frequency.


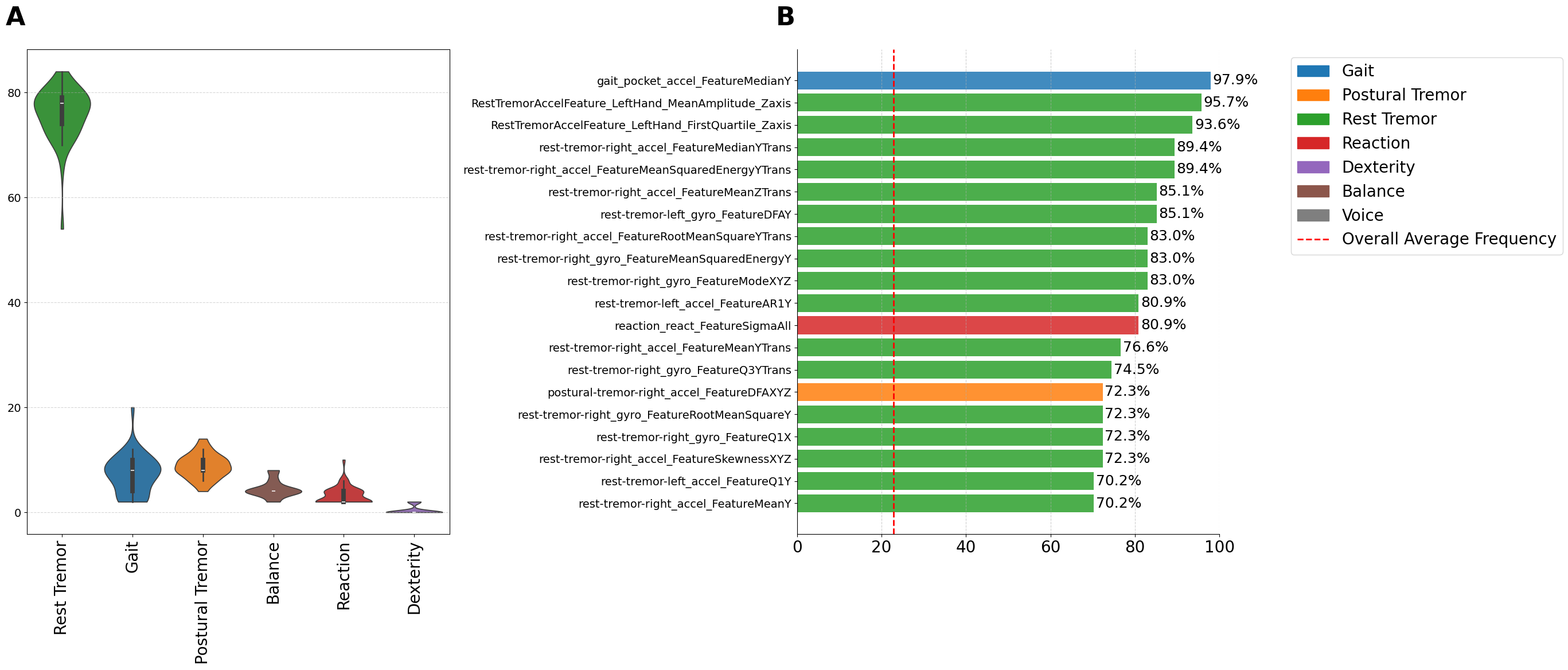


Figure 6. Feature selection summary across smartphone assessments in ON-medication state. **Panel A** reports violin plot of the proportion of features selected per motor task across leave-one-out cross-validation folds. **Panel B** reports horizontal bar plot of the selection frequency (%) of the top 20 individual features; bars are colored by motor task, and the red dashed line indicates the overall average feature selection frequency.


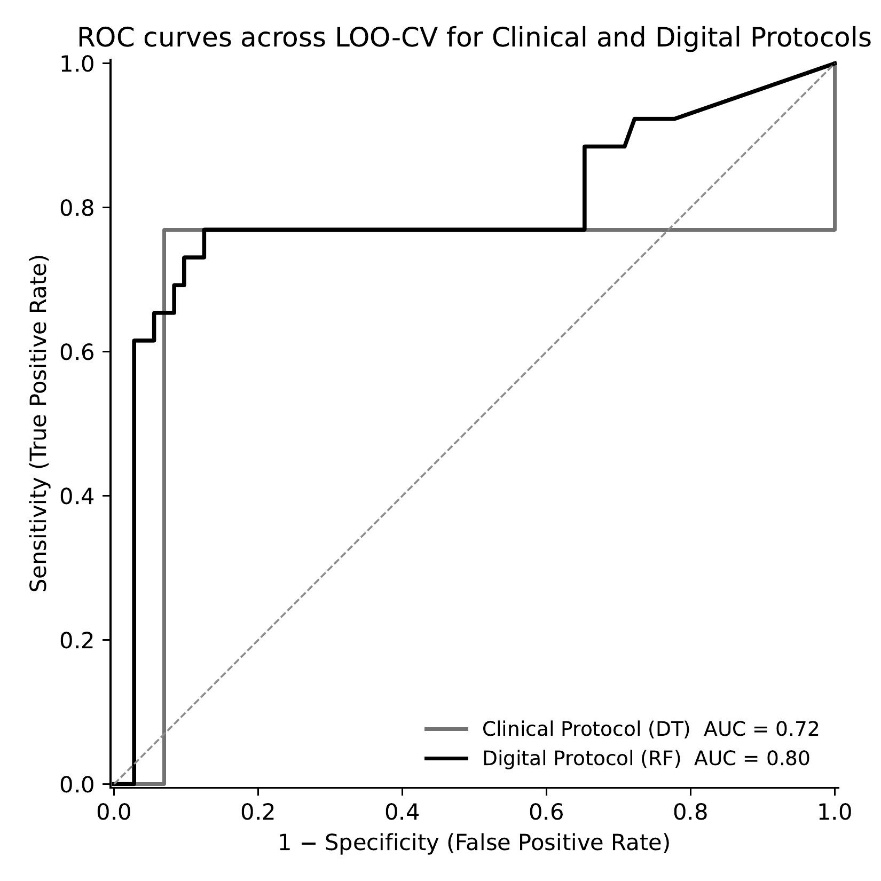


Figure 7. Leave-one-out ROC curves for the Clinical and Digital Protocols on the full longitudinal cohort (n = 98; 26 fast and 72 slow progressors). Both protocols are evaluated on the same 98 patients. The Clinical Protocol (grey) classifies every patient with a depth-1 decision tree trained on baseline OFF-state MDS-UPDRS-3 alone. The Digital Protocol (black) is a dual-pathway design over the same 98 patients. Mean LOO AUCs were 0.72 (95% CI 0.70–0.74) for the Clinical Protocol and 0.80 (95% CI 0.78–0.82) for the Digital Protocol. The step-function appearance of the Clinical Protocol curve reflects the structure of a depth-1 tree on a single continuous predictor: in each LOO fold the tree partitions the training patients into two leaves around an optimal MDS-UPDRS-3 cut-point and outputs the empirical fast-progressor proportion in each leaf as the predicted probability. Because LOO changes the training composition by a single unit per fold, the chosen cut-point and the leaf proportions vary marginally, yielding only a handful of distinct predicted probabilities across the 98 test patients — a low-UPDRS-3 cluster near 0.05–0.09 and a high-UPDRS-3 cluster near 0.79–0.83. The resulting ROC has a small number of corner points and a wide flat segment spanning the score gap between the two leaves, where adjusting the decision threshold cannot change sensitivity or specificity. The Digital Protocol’s smartphone branch produces a continuous probability score that resolves ranking within each MDS-UPDRS-3 sub-population, yielding the smoother curve and the +0.08 AUC gain over the clinical-only model. The thin dashed diagonal represents chance (AUC = 0.50).

| **Inclusion criteria** | 1. Diagnosis of Parkinson’s disease.  2. Hoehn and Yahr stage ≤2.5 in the ON medication state.  3. Between 25 and 80 years of age.  4. On dopaminergic treatment for at least 4 weeks before enrolment.  5. Ability to self-administer, or to arrange carer administration of trial  medication.  6. Documented informed consent to participate. |
| --- | --- |
| **Exclusion criteria** | 1. Diagnosis or suspicion of other cause for Parkinsonism.  2. Patients unable to attend the clinic visits in the practically defined OFF medication state.  3. Body mass index < 18.5  4. Known abnormality on CT or MRI brain imaging considered likely to compromise compliance with trial protocol.  5. Significant cognitive impairment defined by a score < 21 on the Montreal Cognitive Assessment.  6. Concurrent severe depression defined by a score ≥16 on the Patient Health Questionnaire (PHQ-9).  7. Prior intra-cerebral surgical intervention for Parkinson’s disease.  8. Previous participation in one of the following Parkinson’s disease trials.  9. Participation in another clinical trial of a device, drug or surgical treatment within the last 30 days.  10. Previous exposure to exenatide.  11. Impaired renal function with creatinine clearance.  12. History of pancreatitis.  13. Type 1 or Type 2 Diabetes mellitus.  14. Severe gastrointestinal disease (e.g. gastroparesis).  15. Hyperlipidaemia  16. History or family history of medullary thyroid cancer (MTC).  17. Multiple endocrine neoplasia 2 (MEN2) syndrome.  18. Hypersensitivity to any of exenatide's excipients.  19. Females that are pregnant or breastfeeding.  20. WOCBP who are unwilling or unable to use an acceptable method to avoid pregnancy for the entire trial period and up to 3 months after the last dose of trial medication  21. Participants who lack the capacity to give informed consent.  22. Any medical or psychiatric condition or previous conventional/experimental treatment which in the investigator’s opinion compromises the potential participant’s ability to participate |
| **Number of Withdrawal participants** | N = 5 |
| **Withdrawal reason(s)** | - Lost to follow-up, withdrawn from the Exenatide-PD3 Trial (n = 4) - Declined to continue participation, withdrawn from the digital substudy (n=1) |

## Table 1. Inclusion-exclusion criterion and withdrawal information.

| **Subpopulation** | **Category** | **Disease progression** |
| --- | --- | --- |
| **Sex** | Female | Slower |
|  | Male | Faster |
| **Hoehn and Yahr stage** | ≤2 | Slower |
|  | >2 | Faster |
| **Disease duration** | ≤48 months | Slower |
|  | >48 months | Faster |
| **Age** | ≤60 years | Slower |
|  | >60 years | Faster |
| **Age at diagnosis** | ≤60 years | Slower |
|  | >60 years | Faster |
| **Motor phenotypes I** | Tremor-dominant | Slower |
|  | Akinetic-rigid | Faster |
| **Motor phenotypes II** | Tremor-dominant | Slower |
|  | PIGD | Faster |
| **Data-driven motor trajectory** | Slow | Slower |
|  | Fast | Faster |

**Table 2.** **Exploratory subpopulations.** Tremor score was calculated as the mean of MDS-UPDRS-3 items 2.10 (tremor), 3.15a (postural tremor, right hand), 3.15b (postural tremor, left hand), 3.16a (kinetic tremor, right hand), 3.16b (kinetic tremor, left hand), 3.17a (rest tremor amplitude, right upper extremity), 3.17b (rest tremor amplitude, left upper extremity), 3.17c (rest tremor amplitude, right lower extremity), 3.17d (rest tremor amplitude, left lower extremity), 3.17e (rest tremor amplitude, lip/jaw), and 3.18 (constancy of rest tremor). PIGD score was calculated as the sum of baseline MDS-UPDRS-3 items 3.10 (falling), 3.11 (freezing), 3.12 (walking), 3.13 (gait), and 3.14 (postural stability), divided by 5. For Tremor-to-PIGD ratio, subjects were classified as follows: (i) tremor-dominant: Ratio ≥1.15 or PIGD score = 0 with tremor score >0, (ii) PIGD: Ratio ≤0.9 and (iii) intermediate: Ratio >0.9 and <1.15, or both tremor and PIGD scores = 0. For the akinetic-rigid motor phenotype, Akinetic-rigid score was calculated as the mean of MDS-UPDRS-3 items assessing bradykinesia through items 3.4 (finger tapping), 3.5 (hand movements), 3.6 (pronation-supination movements of hands), 3.7 (toe tapping), 3.8 (leg agility), 3.14 (global spontaneity of movement), rigidity through item 3.3, and axial symptoms through items 3.9 (arising from chair), 3.10 (gait), 3.12 (postural stability), 3.13 (posture). Tremor-to-akinetic-rigid ratio was calculated as the ratio of the mean tremor score to the mean akinetic-rigid score. Subjects were classified as follows: (i) akinetic-rigid phenotype: Ratio <0.8, (ii) Tremor-dominant phenotype: Ratio ≥1.0 and (iii) intermediate: Ratio ≥0.8 and <1.0. To derive data-driven slow and fast motor trajectory phenotypes, MDS-UPDRS-3 total scores from each visit were organized as two-dimensional vectors, with disease duration at the visit and MDS-UPDRS-3 total score as coordinates. Patient vector similarity was evaluated using Dynamic Time Warping (DTW) to accommodate varying temporal alignments across participants. A two-class k-means clustering algorithm was then applied to the DTW-derived similarity values, assigning each patient a binary label corresponding to either a slower or faster motor progression trajectory.

| **Num.** | **Question** |
| --- | --- |
| 1 | Do you own a smartphone? |
| 2 | How often do you use smartphone apps? |
| 3 | Before using it, how easy did you think the app would be to use? |
| 4 | Now that you have used it, how easy was the app to use? |
| 5 | How willing would you be to use the app with your nurse / doctor? |
| 6 | How willing would you be to use the app at home to monitor your Parkinson’s outside of clinic? |

## Table 3. Smartphone acceptability questionnaire.

| **Visit (Week)** | **Participants with ≥1 test, N (%)** | **Participants with ≥7 tests, N (%)** | **Participants with ≥14 tests, N (%)** | **Participants with ≥21 tests, N (%)** | **Mean** | **Median** | **Min** | **Q1** | **Q3** | **Max** |
| --- | --- | --- | --- | --- | --- | --- | --- | --- | --- | --- |
| Baseline | 78 (79.6%) | 74 (75.5%) | 70 (71.4%) | 27 (27.6%) | 14.0 | 18.0 | 0 | 8.0 | 21.0 | 23 |
| 24 | 88 (89.8%) | 77 (78.6%) | 65 (66.3%) | 26 (26.5%) | 13.9 | 17.0 | 0 | 9.0 | 20.0 | 26 |
| 48 | 82 (83.7%) | 70 (71.4%) | 62 (63.3%) | 23 (23.5%) | 12.5 | 16.0 | 0 | 3.0 | 20.0 | 34 |
| 72 | 79 (80.6%) | 62 (63.3%) | 54 (55.1%) | 21 (21.4%) | 11.7 | 16.0 | 0 | 1.0 | 19.0 | 25 |
| 96 | 70 (71.4%) | 57 (58.2%) | 42 (42.9%) | 20 (20.4%) | 10.1 | 11.0 | 0 | 0.0 | 21.0 | 23 |

Table 4. Summary of at-home smartphone assessments. Number and proportion of at-home tests completed per participant during the one-week period following each clinic visit.

| **Parameter** | **Coefficient** | **95% CI** | **Z** | **P-Value** | **Adjusted P-Value** |
| --- | --- | --- | --- | --- | --- |
| Intercept | 27.65 | 21.56 to 33.74 | 8.894 | <.001 | <.001 |
| Time (Baseline to Week 96) | 2.78 | -4.63 to 10.19 | 0.735 | 0.463 | 0.891 |
| Sex | 1.29 | -3.45 to 6.03 | 0.533 | 0.594 | 0.972 |
| Age | -0.86 | -6.27 to 4.55 | -0.312 | 0.755 | 0.999 |
| Age at diagnosis | 2.59 | -3.10 to 8.29 | 0.892 | 0.372 | 0.891 |
| Disease duration | 2.38 | -1.94 to 6.70 | 1.081 | 0.28 | 0.891 |
| Hoehn&Yahr | 0.01 | -6.32 to 6.33 | 0.002 | 0.999 | 0.999 |
| Akinetic-rigid | 0.37 | -3.07 to 3.81 | 0.211 | 0.833 | 0.999 |
| PIGD | -2.02 | -5.38 to 1.33 | -1.181 | 0.237 | 0.891 |
| Data-driven cluster | 18.16 | 13.70 to 22.62 | 7.979 | <.001 | <.001 |
| Additional Change (Male vs. Female) | 2.34 | -3.42 to 8.10 | 0.797 | 0.426 | 0.891 |
| Additional Change (>60 vs. ≤60 Years, Age) | -2.29 | -8.87 to 4.29 | -0.682 | 0.495 | 0.891 |
| Additional Change (>60 vs. ≤60 Years, Age at Diagnosis) | 1.59 | -5.33 to 8.52 | 0.451 | 0.652 | 0.978 |
| Additional Change (>48 vs. ≤48 Months) | -0.01 | -5.26 to 5.24 | -0.004 | 0.997 | 0.999 |
| Additional Change (>2 vs. ≤2.0) | -0.05 | -7.74 to 7.65 | -0.012 | 0.991 | 0.999 |
| Additional Change (Akinetic-Rigid vs. Tremor-Dominant) | -2.16 | -6.34 to 2.02 | -1.012 | 0.312 | 0.891 |
| Additional Change (PIGD vs. Tremor-Dominant) | 0.36 | -3.72 to 4.44 | 0.171 | 0.864 | 0.999 |
| Additional Change (Fast vs. Slow) | 8.17 | 2.75 to 13.60 | 2.953 | 0.003 | 0.019 |
| Group Variance | 0.35 |  |  |  |  |

## Table 5. MDS-UPDRS-3 OFF scores Across Predefined and Data-Driven Subpopulations.

| **Parameter** | **Coefficient** | **95% CI** | **Z** | **P-Value** | **Adjusted P-Value** |
| --- | --- | --- | --- | --- | --- |
| Baseline Score (Slower/Slow) | 15.3 | 10.32 to 20.27 | 6.024 | <.001 | <.001 |
| Time (Baseline to Week 96) | 1.18 | -5.05 to 7.41 | 0.372 | 0.71 | 0.799 |
| Sex | 0.39 | -3.48 to 4.26 | 0.198 | 0.843 | 0.843 |
| Age | 3.56 | -0.90 to 8.03 | 1.563 | 0.118 | 0.257 |
| Age at diagnosis | -0.48 | -5.13 to 4.17 | -0.202 | 0.84 | 0.843 |
| Disease duration | -0.69 | -4.22 to 2.84 | -0.384 | 0.701 | 0.799 |
| Hoehn&Yahr | -1.66 | -6.82 to 3.51 | -0.629 | 0.53 | 0.783 |
| Akinetic-rigid | 3.95 | 1.16 to 6.74 | 2.775 | 0.006 | 0.025 |
| PIGD | -2.6 | -5.34 to 0.15 | -1.854 | 0.064 | 0.229 |
| Data-driven cluster | 11.56 | 7.73 to 15.40 | 5.914 | <.001 | <.001 |
| Additional Change (Male vs. Female) | 3.65 | -1.20 to 8.51 | 1.475 | 0.14 | 0.257 |
| Additional Change (>60 vs. ≤60 Years, Age) | -4.97 | -10.56 to 0.62 | -1.741 | 0.082 | 0.245 |
| Additional Change (>60 vs. ≤60 Years, Age at Diagnosis) | 3.5 | -2.33 to 9.33 | 1.177 | 0.239 | 0.392 |
| Additional Change (>48 vs. ≤48 Months) | 1.08 | -3.35 to 5.51 | 0.478 | 0.633 | 0.799 |
| Additional Change (>2 vs. ≤2.0) | 1.9 | -4.57 to 8.36 | 0.575 | 0.566 | 0.783 |
| Additional Change (Akinetic-Rigid vs. Tremor-Dominant) | -2.61 | -6.10 to 0.88 | -1.465 | 0.143 | 0.257 |
| Additional Change (PIGD vs. Tremor-Dominant) | 2.84 | -0.61 to 6.29 | 1.614 | 0.106 | 0.257 |
| Additional Change (Fast vs. Slow) | 12.78 | 7.98 to 17.58 | 5.214 | <.001 | <.001 |
| Group Variance | 0.28 |  |  |  |  |

## Table 6. MDS-UPDRS-3 ON scores across predefined and data-driven subpopulations.

|  |  |  |  | **Placebo** | | | **Exenatide** | | |  | **95% CI** | **Relative difference**  **(RD) ratio** |
| --- | --- | --- | --- | --- | --- | --- | --- | --- | --- | --- | --- | --- |
| **Category** | **Subgroup** | **Trajectory** | **Total**  **n** | **Baseline** | **Week 96** | **Adjusted mean** | **Baseline** | **Week 96** | **Adjusted mean** | **Adjusted means Difference** |  |  |
| **Sex** | Female | Slower | 22 | 20.92 | 22.85 | 1.92 | 17.33 | 19.22 | 1.89 | -0.03 | -2.97 to 2.91 | -0.02 |
|  | Male | Faster | 76 | 24.38 | 27.62 | 3.24 | 21.80 | 30.18 | 8.39 | 5.15 | 3.70 to 6.59 | 1.59 |
| **Disease duration** | ≤48 months | Slower | 39 | 23.81 | 27.05 | 3.24 | 22.64 | 29.67 | 7.03 | 3.79 | 0.46 to 7.13 | 1.17 |
|  | >48 months | Faster | 59 | 23.24 | 25.90 | 2.66 | 20.03 | 27.13 | 7.10 | 4.44 | 3.87 to 5.02 | 1.67 |
| **Age** | ≤60 years | Slower | 39 | 21.33 | 24.14 | 2.81 | 16.61 | 23.83 | 7.22 | 4.41 | 2.80 to 6.03 | 1.57 |
|  | >60 years | Faster | 59 | 25.03 | 28.00 | 2.97 | 23.67 | 30.68 | 7.01 | 4.04 | 2.74 to 5.35 | 1.36 |
| **Age at diagnosis** | ≤60 years | Slower | 60 | 22.65 | 26.00 | 3.35 | 18.45 | 23.59 | 5.14 | 1.78 | 0.60 to 2.97 | 0.53 |
|  | >60 years | Faster | 38 | 24.84 | 27.00 | 2.16 | 24.99 | 35.21 | 10.22 | 8.07 | 6.93 to 9.20 | 3.74 |
| **Hoehn and Yahr stage** | ≤2 | Slower | 87 | 23.74 | 26.12 | 2.37 | 21.21 | 28.09 | 6.88 | 4.51 | 3.17 to 5.85 | 1.90 |
|  | >2 | Faster | 11 | 21.86 | 28.00 | 6.14 | 19.00 | 28.00 | 9.00 | 2.86 | -3.38 to 9.09 | 0.47 |
| **Motor phenotypes I** | Tremor-dominant | Slower | 23 | 24.80 | 27.70 | 2.90 | 22.59 | 29.97 | 7.38 | 4.48 | 3.89 to 5.06 | 1.54 |
|  | Akinetic-rigid | Faster | 64 | 18.43 | 23.00 | 4.57 | 17.30 | 21.28 | 3.99 | -0.59 | -2.94 to 1.76 | -0.13 |
| **Motor phenotypes II** | Tremor-dominant | Slower | 72 | 19.25 | 24.25 | 5.00 | 16.64 | 22.09 | 5.45 | 0.45 | -3.12 to 4.03 | 0.09 |
|  | PIGD | Faster | 20 | 23.94 | 27.00 | 3.06 | 22.16 | 29.08 | 6.92 | 3.86 | 2.63 to 5.08 | 1.26 |
| **Data-driven motor trajectory** | Slower | Slower | 75 | 20.26 | 21.56 | 1.31 | 17.77 | 20.88 | 3.12 | 1.81 | 0.93 to 2.69 | 1.38 |
|  | Faster | Faster | 23 | 34.91 | 43.45 | 8.55 | 30.75 | 49.17 | 18.42 | 9.87 | 8.10 to 11.64 | 1.16 |

## Table 7. Exenatide effects on motor progression as measured by the MDS-UPDRS-3 across the exploratory subpopulations (ON).

|  |  |  |  | **Placebo** | | | **Exenatide** | | |  | **95% CI** | **Relative difference**  **(RD) ratio** |
| --- | --- | --- | --- | --- | --- | --- | --- | --- | --- | --- | --- | --- |
| **Category** | **Subgroup** | **Trajectory** | **Total**  **n** | **Baseline** | **Week 96** | **Adjusted mean** | **Baseline** | **Week 96** | **Adjusted mean** | **Adjusted means**  **Difference** |  |  |
| **Sex** | Female | Slower | 22 | 33.54 | 34.31 | 0.77 | 29.44 | 33.22 | 3.78 | 3.01 | 2.02 to 4.00 | 3.91 |
|  | Male | Faster | 76 | 37.19 | 39.73 | 2.54 | 34.82 | 41.87 | 7.05 | 4.51 | 3.68 to 5.34 | 1.78 |
| **Disease duration** | ≤48 months | Slower | 39 | 34.29 | 36.62 | 2.33 | 33.61 | 40.28 | 6.67 | 4.33 | 0.44 to 8.23 | 1.86 |
|  | >48 months | Faster | 59 | 37.66 | 39.55 | 1.90 | 33.93 | 40.23 | 6.30 | 4.40 | 4.09 to 4.72 | 2.32 |
| **Age** | ≤60 years | Slower | 39 | 34.29 | 35.67 | 1.38 | 32.61 | 40.06 | 7.44 | 6.06 | 4.03 to 8.10 | 4.39 |
|  | >60 years | Faster | 59 | 37.66 | 40.24 | 2.59 | 34.53 | 40.37 | 5.83 | 3.25 | 1.84 to 4.65 | 1.26 |
| **Age at diagnosis** | ≤60 years | Slower | 60 | 36.13 | 38.35 | 2.23 | 31.55 | 37.24 | 5.69 | 3.46 | 2.26 to 4.66 | 1.56 |
|  | >60 years | Faster | 38 | 36.42 | 38.26 | 1.84 | 37.26 | 44.84 | 7.58 | 5.74 | 4.59 to 6.88 | 3.11 |
| **Hoehn and Yahr stage** | ≤2 | Slower | 87 | 35.72 | 37.53 | 1.81 | 33.80 | 40.23 | 6.43 | 4.62 | 3.49 to 5.75 | 2.55 |
|  | >2 | Faster | 11 | 39.43 | 43.14 | 3.71 | 34.00 | 40.50 | 6.50 | 2.79 | -3.95 to 9.52 | 0.75 |
| **Motor phenotypes I** | Tremor-dominant | Slower | 23 | 38.28 | 41.08 | 2.80 | 35.41 | 42.28 | 6.88 | 4.08 | 3.30 to 4.85 | 1.46 |
|  | Akinetic-rigid | Faster | 64 | 26.29 | 28.57 | 2.29 | 30.08 | 34.08 | 4.00 | 1.71 | -0.92 to 4.34 | 0.75 |
| **Motor phenotypes II** | Tremor-dominant | Slower | 72 | 36.92 | 38.92 | 2.00 | 29.82 | 36.91 | 7.09 | 5.09 | 0.48 to 9.70 | 2.55 |
|  | PIGD | Faster | 20 | 34.75 | 37.75 | 3.00 | 34.66 | 40.47 | 5.81 | 2.81 | 1.97 to 3.66 | 0.94 |
| **Data-driven motor trajectory** | Slow | Slower | 72 | 30.86 | 31.65 | 0.78 | 29.09 | 32.71 | 3.63 | 2.84 | 1.51 to 4.18 | 3.63 |
|  | Fast | Faster | 26 | 51.54 | 57.31 | 5.77 | 46.54 | 60.54 | 14.00 | 8.23 | 5.91 to 10.55 | 1.43 |

**Table 8. Exenatide effects on motor progression as measured by the MDS-UPDRS-3 OFF score across the predefined and data-driven exploratory subpopulations (OFF).**

| **Classifier Configuration** | **ON-Medication**  **AUC (95% CI)** | **OFF-Medication**  **AUC (95% CI)** |
| --- | --- | --- |
| Benchmark (MDS-UPDRS-3 only) | 0.54 (0.48-0.59) | 0.53 (0.47-0.59) |
| Smartphone-only (in-clinic data) | 0.73 (0.69-0.76) | 0.78 (0.72-0.82) |
| Multimodal-model (MDS-UPDRS-3 + in-clinic smartphone data) | 0.68 (0.64-0.72) | 0.76 (0.71-0.81) |
| Smartphone-only (in-clinic and at-home smartphone data) | 0.69 (0.63-0.74) | 0.70 (0.67-0.72) |
| Multimodal-model (MDS-UPDRS-3 + in-clinic and at-home smartphone data) | 0.77 (0.71-0.81) | 0.76 (0.72-0.82) |

## Table 9. Predictive Performance of Classifiers for Slow vs Fast Motor Progression Trajectories at Baseline. Model performance was assessed using a Bayesian estimate of the area under the receiver operating characteristic curve (AUC). This approach calculates the probability that a randomly selected positive case has a higher predicted score than a randomly selected negative case. 95% credible intervals for the AUC were derived from a Beta posterior distribution, providing a robust measure of uncertainty without relying on resampling techniques.

| **Smartphone assessment** | **Mean** | **std** | **Median** | **Min** | **Q1** | **Q3** | **Max** |
| --- | --- | --- | --- | --- | --- | --- | --- |
| Rest Tremor | 39.7 | 5.51 | 40.0 | 28.0 | 36.0 | 44.0 | 52.0 |
| Gait | 24.5 | 3.88 | 26.0 | 16.0 | 22.0 | 27.0 | 30.0 |
| Postural Tremor | 23.2 | 6.40 | 22.0 | 12.0 | 19.0 | 30.0 | 34.0 |
| Balance | 7.0 | 2.39 | 6.0 | 2.0 | 6.0 | 8.0 | 14.0 |
| Reaction | 4.0 | 3.06 | 4.0 | 0.0 | 2.0 | 6.0 | 12.0 |
| Dexterity | 1.5 | 1.14 | 2.0 | 0.0 | 0.0 | 2.0 | 4.0 |
| Voice | 0.0 | 0.0 | 0.0 | 0.0 | 0.0 | 0.0 | 0.0 |

Table 10. Task-level frequency table (OFF-medication state). Summarizes the repartition of feature families per fold, i.e., the proportion (%) of selected features from each smartphone task in each fold.

| **Smartphone assessment** | **Mean** | **std** | **Median** | **Min** | **Q1** | **Q3** | **Max** |
| --- | --- | --- | --- | --- | --- | --- | --- |
| Rest Tremor | 75.9 | 5.02 | 78.0 | 54.0 | 74.0 | 79.0 | 84.0 |
| Gait | 7.2 | 3.64 | 8.0 | 2.0 | 4.0 | 10.0 | 20.0 |
| Postural Tremor | 8.7 | 2.30 | 8.0 | 4.0 | 8.0 | 10.0 | 14.0 |
| Balance | 4.4 | 1.67 | 4.0 | 2.0 | 4.0 | 4.0 | 8.0 |
| Reaction | 3.2 | 1.64 | 2.0 | 2.0 | 2.0 | 4.0 | 10.0 |
| Dexterity | 0.4 | 0.79 | 0.0 | 0.0 | 0.0 | 0.0 | 2.0 |
| Voice | 0.0 | 0.0 | 0.0 | 0.0 | 0.0 | 0.0 | 0.0 |

Table 11. Task-level frequency table (ON-medication state). Summarizes the repartition of feature families per fold, i.e., the proportion (%) of selected features from each smartphone task in each fold.

| **Feature** | **Motor Test** | **Frequency (%)** |
| --- | --- | --- |
| GaitAccelerometerFeature_DFA_YaxisTransformed | Gait | 100.0 |
| GaitGyroscopeFeature_1stQuartile_XYZaxis | Gait | 97.9 |
| PosturalTremorAccelFeature_LeftHand_RootMeanSquare_Xaxis | Postural Tremor | 97.9 |
| RestTremorAccelFeature_LeftHand_FirstQuartile_Zaxis | Rest Tremor | 95.7 |
| RestTremorGyroFeature_LeftHand_AR1coeff_YaxisTransformed | Rest Tremor | 93.6 |
| GaitAccelerometerFeature_DFA_XYZaxis | Gait | 87.2 |
| RestTremorAccelFeature_LeftHand_MeanAmplitude_Zaxis | Rest Tremor | 82.9 |
| RestTremorAccelFeature_RightHand_LempelZivMeasure_Zaxis | Rest Tremor | 82.9 |
| RestTremorGyroFeature_LeftHand_CrossEntropy_XYaxis | Reaction | 76.5 |
| ReactionTimeFeature_DiffInPressReleaseReactionTimes | Rest Tremor | 76.5 |
| GaitAccelerometerFeature_DominantFrequency_Yaxis | Gait | 74.5 |
| RestTremorGyroFeature_RightHand_AR1coeff_XYZaxis | Postural Tremor | 74.5 |
| RestTremorGyroFeature_LeftHand_LempelZivMeasure_Zaxis | Rest Tremor | 74.5 |
| PosturalTremorAccelFeature_LeftHand_RootMeanSquare_XYZaxis | Rest Tremor | 74.5 |
| GaitAccelerometerFeature_Skewness_Zaxis | Gait | 70.2 |
| GaitGyroscopeFeature_SpectralFlux_Zaxis | Gait | 70.2 |
| RestTremorGyroFeature_LeftHand_Skewness_ZaxisTransformed | Rest Tremor | 70.2 |
| GaitAccelerometerFeature_DFA_Yaxis | Gait | 63.8 |
| GaitGyroscopeFeature_Mode_XYZaxis | Gait | 63.8 |
| GaitAccelerometerFeature_MutualInformation_YZaxis | Gait | 61.7 |

Table 12. Feature frequency table (OFF-medication state). Top-20 most frequently selected features across leave-one-out cross-validation. The table reports each feature, its corresponding motor task assessment, and the frequency (%) of selection across all folds.

| **Feature** | **Motor Test** | **Frequency (%)** |
| --- | --- | --- |
| GaitAccelerometerFeature_Median_Yaxis | Gait | 97.8 |
| RestTremorAccelFeature_LeftHand_MeanAmplitude_Zaxis | Rest Tremor | 95.7 |
| RestTremorAccelFeature_LeftHand_FirstQuartile_Zaxis | Rest Tremor | 93.6 |
| RestTremorAccelFeature_RightHand_MeanSquaredEnergy_YaxisTransformed | Rest Tremor | 89.3 |
| RestTremorAccelFeature_RightHand_Median_YaxisTransformed | Rest Tremor | 89.3 |
| RestTremorGyroFeature_LeftHand_DFA_Yaxis | Rest Tremor | 85.1 |
| RestTremorAccelFeature_RightHand_Mean_ZaxisTransformed | Rest Tremor | 85.1 |
| RestTremorGyroFeature_RightHand_Mode_XYZaxis | Rest Tremor | 82.9 |
| RestTremorGyroFeature_RightHand_MeanSquaredEnergy_Yaxis | Rest Tremor | 82.9 |
| RestTremorAccelFeature_RightHand_MeanSquaredEnergy_YaxisTransformed | Rest Tremor | 82.9 |
| ReactionTimeFeature_exGaussAnalysisSigmaParameter | Reaction | 80.8 |
| RestTremorAccelFeature_LeftHand_AR1coeff_Yaxis | Rest Tremor | 80.8 |
| RestTremorAccelFeature_RightHand_Mean_YaxisTransformed | Rest Tremor | 76.5 |
| RestTremorGyroFeature_RightHand_ThirdQuartile_YaxisTransformed | Rest Tremor | 74.4 |
| PosturalTremorAccelFeature_RightHand_DFA_XYZaxis | Postural Tremor | 72.3 |
| RestTremorGyroFeature_RightHand_FirstQuartile_Xaxis | Rest Tremor | 72.3 |
| RestTremorAccelFeature_RightHand_Skewness_XYZaxis | Rest Tremor | 72.3 |
| RestTremorGyroFeature_RightHand_RootMeanSquare_Yaxis | Rest Tremor | 72.3 |
| RestTremorAccelFeature_RightHand_Mean_Yaxis | Rest Tremor | 70.2 |
| RestTremorAccelFeature_LeftHand_FirstQuartile_Yaxis | Rest Tremor | 70.2 |

Table 13. Feature frequency table (ON-medication state). Top-20 most frequently selected features across leave-one-out cross-validation. The table reports each feature, its corresponding motor task assessment, and the frequency (%) of selection across all folds.

| **Classifier Configuration** | **ON-Medication**  **AUC (95% CI)** | **OFF-Medication**  **AUC (95% CI)** |
| --- | --- | --- |
| Benchmark (MDS-UPDRS-3 total scores only) | 0.49 (0.45-0.53) | 0.51 (0.44-0.57) |
| Smartphone-only (in-clinic data) | 0.45 (0.40-0.51) | 0.39 (0.32-0.45) |
| Smartphone-only (in-clinic + at-home data) | 0.46 (0.42-0.58) | 0.48 (0.40-0.57) |

**Table 14. Placebo vs Exenatide groups prediction.**

| **Classifier Configuration** | **ON-Medication**  **AUC (95% CI)** | **OFF-Medication**  **AUC (95% CI)** |
| --- | --- | --- |
| Benchmark Clinical Protocol | 0.67 (0.64-0.70) | 0.72 (0.70-0.74) |
| Digital Protocol | 0.74 (0.70-0.77) | 0.80 (0.79-0.82) |

## Table 15. Comparison of Benchmark Clinical vs Digital Protocols for Predicting Motor Trajectories at Baseline.

| **Protocol** | AUC (95% CI) | Sensitivity | Specificity | PPV | NPV | Brier |  |  |
| --- | --- | --- | --- | --- | --- | --- | --- | --- |
| Clinical Protocol | 0.73 (0.70–0.75) | 0.82 | 0.89 | 0.75 | 0.92 | 0.13 |  |  |
| Digital Protocol | 0.80 (0.77–0.81) | 0.73 | 0.87 | 0.70 | 0.89 | 0.14 |  |  |
| Null reference | 0.50 |  |  |  |  | 0.20 |  |  |

## Table 16. Threshold-based performance metrics at the Youden’s J-optimal cut-off, with calibration summary (Brier score) and a null-model reference .

| **k** | **silhouette** | **Gap statistic** |
| --- | --- | --- |
| 2 | 0.502 | 1.52 |
| 3 | 0.417 | 1.94 |
| 4 | 0.343 | 2.15 |
| 5 | 0.285 | 2.20 |

## Table 17.  Label agreement under an alternative distance metric (MDS-UPDRS-3 OFF).

| Distance metric | Same fast/slow label as DTW | Distance metric |
| --- | --- | --- |
| Euclidean | 89 / 98 (91%) | Euclidean |

## Table 18.  Label agreement under an alternative distance metric (MDS-UPDRS-3 OFF).

| Classifier | ARI vs DTW cluster |
| --- | --- |
| Linear-slope median split | 0.07 |
| Endpoint minus baseline median split | 0.11 |

## Table 19.  Agreement (ARI) between simple median-split classifiers and the DTW cluster.

| Predictor | Standardised coefficient | Univariate AUC |
| --- | --- | --- |
| Baseline OFF MDS-UPDRS-3 | 2.03 | 0.86 |
| Per-patient linear slope | 1.36 | 0.67 |
| Joint model |  | 0.95 |

## Table 20.  Standardised coefficients and univariate AUCs from the logistic-regression decomposition of cluster membership on baseline severity and per-patient slope.

| Term | Estimate (per visit) | 95% CI |
| --- | --- | --- |
| Time slope (slow placebo reference) | −0.13 | (−0.73 to 0.47) |
| Time × exenatide | +0.42 | (−0.42 to 1.26) |
| Time × fast cluster | +2.21 | (1.02 to 3.40) |
| Time × exenatide × fast cluster (interaction) | +1.42 | (−0.21 to 3.04) |

## Table 21.  Treatment × cluster × time interaction estimates from the linear mixed model.

| Covariate | Exenatide (n = 13) | Placebo (n = 13) |
| --- | --- | --- |
| Age (years) | 65.4 (60.1 to 70.7) | 63.6 (59.4 to 67.8) |
| Sex (% male) | 85% (58 to 96%) | 85% (58 to 96%) |
| Disease duration (months) | 60.0 (38.0 to 82.0) | 60.9 (41.4 to 80.4) |
| Baseline MDS-UPDRS-3 OFF | 46.5 (41.2 to 51.8) | 51.5 (47.4 to 55.6) |
| Baseline MDS-UPDRS-3 ON | 29.3 (21.3 to 37.3) | 34.8 (30.5 to 39.2) |

## Table 22. Fast-progressor demographics.Secondary analysis

## ON-medication state sensitivity analysis

### **Identification of fast and slow progressors**

Changes in MDS-UPDRS-3 scores from Baseline to Week 96 in the ON condition varied across predefined and data-driven subpopulations (**Table 6**). Among predefined exploratory subpopulations, no significant differences in score changes were observed between Slower and Faster progressors after false discovery rate (FDR) correction. In contrast, the data-driven approach effectively identified slower and faster progressors. At Baseline, *fast* progressors had MDS-UPDRS-3 scores 11.56 points higher (95% CI, 7.73-15.40; P < .001) than slow progressors. From Baseline to Week 96, *fast* progressors exhibited an additional increase of 12.78 points (95% CI, 7.98-17.58; P < .001; adjusted P < .001) compared to *slow* progressors, remaining significant after FDR correction.

### **Motor Sign Progression Between Treatment and Control Groups Within Subpopulations**

Adjusted mean changes in MDS-UPDRS-3 scores from Baseline to Week 96 in the ON state varied across subpopulations, with the largest treatment differences observed in data-driven fast progressors (**Table 7**). Among predefined subpopulations, exenatide vs placebo differences in score changes ranged from -0.59 (95% CI, -2.94 to 1.76; RD, -0.13) for Akinetic-rigid to 8.07 (95% CI, 6.93-9.20; RD, 3.74) for Age at diagnosis, >60 years. In the data-driven subpopulations, *slow* progressors had an adjusted mean change of 1.31 with placebo vs 3.12 with exenatide (difference, 1.81 [95% CI, 0.93-2.69; RD, 1.38]), while *fast* progressors showed 8.55 with placebo vs 18.42 with exenatide (difference, 9.87 [95% CI, 8.10-11.64; RD, 1.16]), the largest treatment effect observed. Baseline scores for slow progressors were 20.26 (placebo) and 17.77 (exenatide), increasing to 21.56 and 20.88, respectively, while fast progressors, with higher baseline severity (34.91 placebo, 30.75 exenatide), rose to 43.45 and 49.17, respectively.

### **Prediction of Data-Driven Fast vs. Slow Motor Progression Trajectories**

### **Baseline Prediction Using Clinical and Smartphone Data**

Predictive models classified data-driven fast vs. slow progressors (n = 47) at Baseline using MDS-UPDRS-3 scores and smartphone-derived data **(Table 9**). The benchmark classifier (MDS-UPDRS-3 only) yielded AUCs of 0.54. Smartphone-only in-clinic data improved AUCs to 0.73. Adding in-clinic MDS-UPDRS-3 scores to smartphone data resulted in an AUC of 0.68. Smartphone-only data (in-clinic and at-home) achieved a comparable AUC of 0.69. A multimodal-model integrating MDS-UPDRS-3 with in-clinic and at-home smartphone data produced an AUC of 0.77.

### **Complementary results on smartphone utility**

Regarding alignment with the primary Exenatide-PD3 trial outcome, each of the benchmark classifiers (MDS-UPDRS-3 only), Smartphone-only classifier with in-clinic data and Smartphone-only classifier with in-clinic and at-home data trained to differentiate placebo from exenatide participants yielded poor AUCs of around 0.5 (**Table 15**).

Regarding the utility of a digital protocol framework, the digital protocol, integrating MDS-UPDRS-3 (n = 98) and smartphone data (n = 79) when available, outperformed the benchmark clinical protocol (MDS-UPDRS-3 only, n = 98) in predicting slow vs. fast motor trajectories at Baseline, achieving an AUC of 0.74 vs 0.67.

### **Feature Selection Analysis for ON-Medication State**

Feature selection for smartphone-based models in the ON-medication state was conducted using in-clinic data across leave-one-out cross-validation (LOO-CV) folds, described in terms of frequency of occurrence and motor task repartition, to enable participant comparison (**Supplement Figure 6**, **Table 11**, **Table 13**). Across LOO-CV folds, rest tremor features were most frequently selected (mean 75.9%, SD 5.02%), followed by postural tremor (mean 8.7%, SD 2.30%), gait (mean 7.2%, SD 3.64%), balance (mean 4.4%, SD 1.67%), reaction (mean 3.2%, SD 1.64%), and dexterity (mean 0.4%, SD 0.79%), with no voice features selected (**Table 11**). Among the top 20 features, 17 were rest tremor-related, 1 was postural tremor-related, 1 was gait-related, 1 was reaction-related, with selection frequencies ranging from 70.2% to 97.8% (**Table 13**, **Figure 6**). Compared to the OFF-medication state, where rest tremor and gait features were equally represented (8 each of top 20; mean 39.7% and 24.5%, respectively; **Table 10**, **Table 12**), the ON state showed a marked dominance of rest tremor features and fewer gait features, likely reflecting medication-related suppression of gait impairments (**Figure 5**).

## Post-hoc analysis

## Clustering analysis

## 1. Choice of k.

## k = 2 was set a priori for methodological consistency with the binary fast-vs-slow progressor framework established by Pagano et al. (2024)^1^. To analyse the empirical support for this choice across alternative values of k, the silhouette score and the gap statistic^2^ were computed for k ∈ {2, 3, 4, 5} on the disease-duration-aligned MDS-UPDRS-3 OFF visit matrix. The silhouette for an observation i is defined as $\boldsymbol{s}\left( \boldsymbol{i} \right)\boldsymbol{=}\frac{\left( \boldsymbol{b}\left( \boldsymbol{i} \right)\boldsymbol{- a}\left( \boldsymbol{i} \right) \right)}{\boldsymbol{max(a(i), b(i))}}$, where $\boldsymbol{a(i)}$ is the mean intra-cluster distance and $\boldsymbol{b(i)}$ is the mean nearest-other-cluster distance; the silhouette of a clustering is the mean of $\boldsymbol{s(i)}$ over all observations and lies in [−1, 1] with higher values indicating better separation. The gap statistic compares the log within-cluster dispersion log $\boldsymbol{W}_{\boldsymbol{k}}$ of the observed clustering with its expectation under reference uniform distributions: $\boldsymbol{Gap}\left( \boldsymbol{k} \right)\boldsymbol{= E.[log}\boldsymbol{W}_{\boldsymbol{k}}\boldsymbol{] - log}\boldsymbol{W}_{\boldsymbol{k}}$.

## 2. Cluster stability. Four complementary analyses were conducted.

## (i) Bootstrap stability. Cluster stability under participant resampling was assessed over 1000 bootstrap iterations. At each iteration, participants were sampled with replacement from the cohort, and the DTW-based k-means clustering (k = 2) was refitted. Agreement between each resampled clustering and the original assignment was quantified by the Adjusted Rand Index (ARI)^3^, which corrects the pair-counting Rand Index for chance and is bounded in [−1, 1] with 1 indicating identical labellings, 0 random agreement, and negative values worse-than-chance agreement. The ARI is reported as the median across iterations.

## (ii) Distance-metric sensitivity. The cohort was re-clustered using the Euclidean metric in place of DTW. The proportion of patients receiving the same fast/slow label as under the published DTW assignment was reported.

## (iii) Comparison with simpler progression definitions. Two median-split classifiers were defined: (a) the per-patient ordinary least-squares slope of MDS-UPDRS-3 OFF across visits, and (b) the visit-5 minus visit-1 difference. Each was compared with the DTW cluster assignment using the Adjusted Rand Index (ARI).

## (iv) Baseline-versus-rate decomposition. A logistic regression of cluster membership on standardised baseline MDS-UPDRS-3 OFF and standardised per-patient linear slope was fitted to quantify the relative contribution of baseline severity and rate of change. Standardised regression coefficients were reported, together with the area under the receiver-operating-characteristic curve (AUC) for each univariate predictor and for the joint model.

## Clustering results

Two analyses are reported below: (1) empirical support across alternative values of k for the a priori choice of k = 2, and (2) the cluster stability across resampling, distance metric, alternative progression definitions, and baseline-versus-rate decomposition.

1. *Choice of k* (**Table 17**)*.*

The silhouette score was highest at k = 2 and declined monotonically for higher values, identifying k = 2 as the partition with the strongest within-data separation. The gap statistic increased monotonically with k and did not show a sharp elbow.

1. *Cluster stability* (**Table 18**)*.*

*(a) Bootstrap stability.* Across 1000 participant-resampled bootstrap iterations, the median Adjusted Rand Index against the published cluster assignment was 0.67, indicating substantial stability under resampling.

*(b) Distance-metric sensitivity.* Re-clustering under the Euclidean metric agreed with the published DTW assignment for 89 of 98 patients (91% identical labels), indicating that the cluster assignment is largely insensitive to the choice of distance metric within this family of methods.

*(c) Comparison with simpler progression definitions* (**Table 19**)*.* Median-split classifiers based on the per-patient linear slope or on the visit-5 minus visit-1 difference recovered the DTW cluster only weakly, indicating that the DTW-based clustering captures trajectory-shape information beyond what is encoded in simple slope or endpoint summaries.

*(d) Baseline-versus-rate decomposition (***Table 20***).* Baseline MDS-UPDRS-3 OFF contributes a strong univariate signal for cluster membership (AUC 0.86), consistent with the reported association between baseline motor severity and subsequent progression rate in Parkinson’s disease. Per-patient linear slope contributes additional, complementary information (univariate AUC 0.67); the joint logistic-regression model containing both predictors yields AUC 0.95.

- **Threshold-based performance metrics and calibration summaries**

Threshold-based performance metrics and calibration summaries are reported below for the two evaluated prediction protocols. The Clinical Protocol uses a Decision Tree trained on baseline clinical variables; the Digital Protocol uses a Random Forest trained on smartphone-derived features. All values are derived from the pooled LOO-CV predicted probabilities; AUC is shown alongside its Bayesian 95% CI, and the Brier score serves as a calibration summary (lower is better). The Brier score of a null model predicting class prevalence is included as a reference.

Both protocols exhibit informative discrimination (**Table 16**), with AUC values above 0.7 and Brier scores below the null-model reference. The Digital Protocol shows higher discrimination (AUC 0.80, against 0.73 for the Clinical Protocol), consistent with the added value of smartphone-derived features. At the Youden’s J-optimal threshold, sensitivity, specificity, PPV, and NPV remain broadly comparable between the two protocols, indicating that the digital and clinical assessments converge on a consistent stratification of fast versus slow progressors while operating on complementary input modalities.

- **Fast-progressor subgroup analysis**

A descriptive linear mixed model (**Table 21**) was fitted on all five MDS-UPDRS-3 OFF visits (baseline, weeks 24, 48, 72, 96), modelling differential progression via time-by-group interactions: a time slope, a time × treatment interaction, a time × cluster interaction, and a time × treatment × cluster interaction, with a random per-patient intercept. The interaction estimate is interpreted descriptively. Descriptive demographics were provided (**Table 22**).

The three-way interaction directionally consistent with the descriptive observation that exenatide-arm fast progressors exhibited greater apparent worsening than placebo-arm fast progressors. The cumulative differential effect at week 96 (t = 4) is +5.7 points (95% CI −0.83 to +12.16, p = 0.087), aligning in magnitude with the implicit difference of adjusted-mean changes reported in main Table 3 (fast 8.23 minus slow 2.84 = 5.39).

**References**

1. Pagano, G. *et al.* Prasinezumab slows motor progression in rapidly progressing early-stage Parkinson’s disease. *Nat. Med.* **30**, 1096–1103 (2024).

2. Tibshirani, R., Walther, G. & Hastie, T. Estimating the Number of Clusters in a Data Set Via the Gap Statistic. *J. R. Stat. Soc. Ser. B Stat. Methodol.* **63**, 411–423 (2001).

3. Hubert, L. & Arabie, P. Comparing partitions. *J. Classif.* **2**, 193–218 (1985).
